# Self-assembly and spectroscopic fingerprints of photoactive pyrenyl tectons on *h*BN/Cu(111)

**DOI:** 10.3762/bjnano.11.130

**Published:** 2020-09-29

**Authors:** Domenik M Zimmermann, Knud Seufert, Luka Ðorđević, Tobias Hoh, Sushobhan Joshi, Tomas Marangoni, Davide Bonifazi, Willi Auwärter

**Affiliations:** 1 Physics Department E20, Technical University of Munich, James-Franck-Straße 1, D-85748 Garching, Germany; 2 The School of Chemistry, Cardiff University, UK-CF10 3AT Cardiff, United Kingdom; 3 Department of Chemical and Pharmaceutical Sciences, University of Trieste, I-34127 Trieste, Italy; 4 Institute of Organic Chemistry, Faculty of Chemistry, University of Vienna, Währinger Str. 38, 1090 Vienna, Austria

**Keywords:** electronic structure, hexagonal boron nitride, optical properties, pyrene, self-assembly

## Abstract

The controlled modification of electronic and photophysical properties of polycyclic aromatic hydrocarbons by chemical functionalization, adsorption on solid supports, and supramolecular organization is the key to optimize the application of these compounds in (opto)electronic devices. Here, we present a multimethod study comprehensively characterizing a family of pyridin-4-ylethynyl-functionalized pyrene derivatives in different environments. UV–vis measurements in toluene solutions revealed absorption at wavelengths consistent with density functional theory (DFT) calculations, while emission experiments showed a high fluorescence quantum yield. Scanning tunneling microscopy (STM) and spectroscopy (STS) measurements of the pyrene derivatives adsorbed on a Cu(111)-supported hexagonal boron nitride (*h*BN) decoupling layer provided access to spatially and energetically resolved molecular electronic states. We demonstrate that the pyrene electronic gap is reduced with an increasing number of substituents. Furthermore, we discuss the influence of template-induced gating and supramolecular organization on the energies of distinct molecular orbitals. The selection of the number and positioning of the pyridyl termini in tetrasubstituted, *trans*- and *cis*-like-disubstituted derivatives governed the self-assembly of the pyrenyl core on the nanostructured *h*BN support, affording dense-packed arrays and intricate porous networks featuring a kagome lattice.

## Introduction

Atomic-level control of molecular materials at interfaces is crucial to fully exploit the materials’ potential in electronic, optoelectronic, spintronic, and sensing applications [1–2]. Specifically, the effects of adsorption, conformation, and supramolecular organization on the resulting electronic and optical properties of molecular tectons and the respective assemblies must be comprehensively characterized [3–6]. Diverse self-assembly protocols have been extensively explored on metal substrates, and organic–metal interfaces have been analyzed in great detail [7–8]. In many cases, molecule–metal interactions can adversely affect the intrinsic electronic characteristics of molecular adsorbates and quench the optical properties [9–13]. Consequently, recent studies aiming to characterize the relation of adsorption, supramolecular organization, and electronic and optical properties in organic layers relied on bulk insulator supports [14–16]. As a promising alternative to bulk insulators, ultrathin dielectric films can act as decoupling layers but maintain the possibility to perform STM and STS measurements [17]. Atomically-thin *h*BN sheets attracted considerable interest as such spacer layers [18] and can promote site-dependent decoupling and adsorption [19–20], yielding access to optical transitions [21] as well as allowing for orbital-resolved STM imaging [19,21–23]. For instance, *h*BN/Cu(111) [24–27] features a work function template with a moiré superstructure: Depending on the registry of the layer and substrate atoms, the surface is divided in areas of low and high local work function, denoted as “pores” and “wires”, respectively [28–31]. In recent years, our group and others used *h*BN/Cu(111) to guide the self-assembly of porphyrins [28,32–33], decouple perylenetetracarboxylic dianhydride (PTCDA) aggregates [34], study interfacial charge transfer in binary phthalocyanine arrays [35], probe vibronic conductance in oligophenylenes [36], and control the charge state of F_16_CoPc [37]. Studies focusing on the preparation of coordination networks [38], wires of polycyclic aromatic hydrocarbons [39], and graphene patches [40–41] were also performed on *h*BN/Cu(111). However, advanced supramolecular architectures, such as organic porous networks, have not been reported on either *h*BN/Cu(111) or other metal-supported *h*BN monolayers so far, in contrast to graphene [42–44] or bulk *h*BN [14,45–46].

Pyrene derivatives are excellent candidates to study the interplay of functionalization and supramolecular organization as the planar polycyclic aromatic core provides an extended π-system that can be readily substituted at distinct positions [47–54]. With prominent fluorescence properties, pyrene is often considered the "fruitfly of photochemists", and several materials have been prepared for applications in optoelectronic devices and organic electronics [47]. On metal surfaces under ultrahigh vacuum (UHV), unsubstituted [55] as well as functionalized pyrene derivatives [9,48,56–64] were employed as versatile tectons to engineer supramolecular [9,48,55–59,61–64] and covalent architectures [56,58–60,64]. On two-dimensional materials, pyrene serves as an anchor for noncovalent functionalization, e.g., to develop graphene platforms to be used in sensing applications [65–67] and to employ *h*BN monolayers for capturing aromatic organic pollutants [68]. On bulk insulators, it was, for instance, demonstrated how the optical properties of an adsorbed bispyrene derivative relate to the structural order of the assemblies [15].

In this paper, we address the effects of chemical substituents on the electronic and self-assembly properties of pyrene derivatives on a *h*BN/Cu(111) substrate. To this end, pyrenyl derivatives bearing four and two pyridin-4-ylethynyl substituents have been used to steer and control the self-assembly on *h*BN/Cu(111), including the formation of dense-packed arrays and intricate kagome networks. The resulting structures deviate in part from the assemblies previously studied on Ag(111) [48]. Additionally, the *h*BN decoupling layer allows the determination of the electronic properties of the pyrene adsorbates by STM and STS", and the comparison with the gaps estimated by theoretical simulations in vacuum and by UV-vis spectroscopies in solution. Remarkably, the electronic states of the pyrene adsorbates near the Fermi level, probed at the submolecular level via STM and STS, e.g., reveal to be close to the gas-phase-like frontier orbitals.

The electronic landscape of the *h*BN/Cu(111) template induces a periodic modulation of the electronic structure of the pyrene films at the single digit nanometer scale. The on-surface STM/STS experiments, the photophysical characterization in solution, and the DFT modeling (in vacuum and with toluene solvation) evidence a reduction of the molecular gap when proceeding from di- to tetrasubstituted pyrene derivatives, but with effects that are different depending on the chemical surrounding.

## Results

### Structural and electronic properties in vacuum

**Figure 1:**
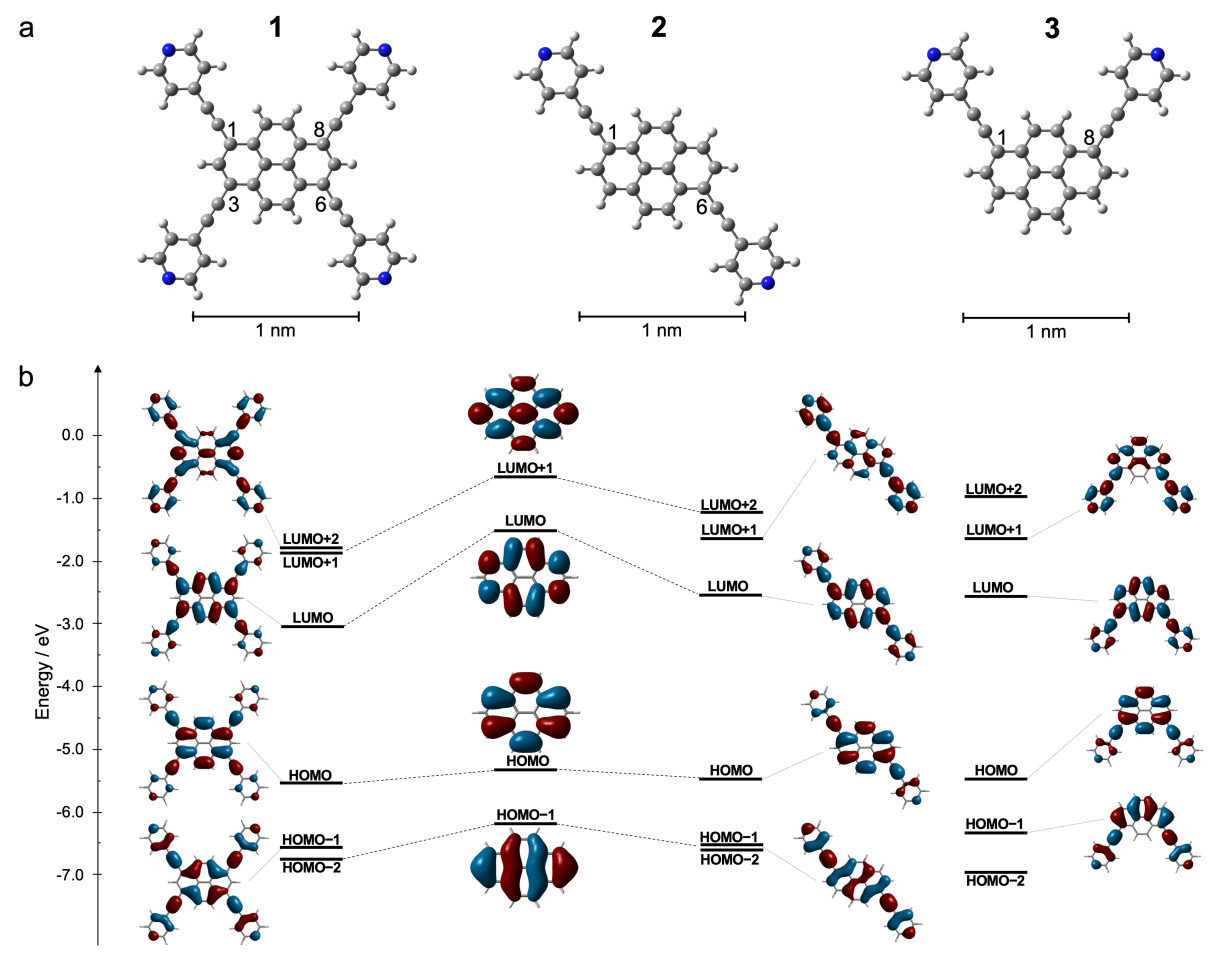
Functionalized pyrene derivatives investigated in this work (DFT-optimized geometries in the gas phase). a) Structure of 1,3,6,8-tetrakis(pyridin-4-ylethynyl)pyrene (**1**, *tetra*), 1,6-bis(pyridin-4-ylethynyl)pyrene (**2**, *trans-*like), and 1,8-bis(pyridine-4-ylethynyl)pyrene (**3**, *cis-*like). Element colors: N (blue), C (grey), H (white). b) Schematic drawing of the frontier Kohn–Sham orbitals for the tetra-, *trans*-like-, and *cis*-like-pyridyl–ethynyl-substituted pyrene derivatives **1**, **2**, and **3**, together with an orbital correlation diagram in comparison to the molecular orbitals (MOs) for pyrene itself, at the B3LYP/6-31G** level of theory.

The pyrenyl derivatives studied in this work are shown in Figure 1a and have been prepared following previous synthetic protocols [48]. The highest molecular symmetry is given by the tetrasubstituted species **1**, namely 1,3,6,8-tetrakis(pyridin-4-ylethynyl)pyrene. Reducing the number of substituents to two reduces the molecular symmetry, and concomitantly introduces interesting new properties, leading to prochiral *trans*-like-substituted 1,6-bis(pyridin-4-ylethynyl)pyrene (**2**) and polar *cis*-like-substituted 1,8-bis(pyridin-4-ylethynyl)pyrene (**3**).

To evaluate the effect of the substitution on the electronic properties of the pyrene core, DFT calculations were performed (B3LYP/6-31G** level of theory, in vacuum). The frontier Kohn-Sham orbitals of pyrene and the di- and tetrasubstituted (pyridin-4-ylethynyl)pyrenes **1**, **2**, and **3** are shown in Figure 1b (see also Figures S1–S5, Supporting Information File 1). The computations revealed that the pyrenes have large orbital coefficients at the 1-, 3-, 6-, and 8-positions, with the nodal plane going through the 2- and 7-positions (Figure 1) [69–74]. As a consequence of this spatial distribution, the orbital interactions between the pyrene and the pyridin-4-ylethynyl MOs had a stabilizing effect on the highest occupied (HOMO) and lowest unoccupied molecular orbital (LUMO) energy levels. While the HOMO stabilization played only a small part, it was the considerable lowering of the LUMO energy levels that governed the shrinking of the HOMO–LUMO gap upon the derivatization with pyridin-4-ylethynyl groups. The picture of the orbital interactions was similar in the di- and tetrapyrenyl derivatives, with the HOMO–LUMO gap being influenced mostly by the number of substituents. The molecular gap of the tetrasubstituted pyrene **1** (2.54 eV) became narrower than that of the disubstituted pyrenes **2** and **3** (2.95 eV and 2.94 eV, respectively). This was in accordance with our experimental findings (vide infra) and previous literature reports [70–71,74].

### Molecular self-assembly on *h*BN/Cu(111)

#### Tetrasubstituted pyrene

**Figure 2:**
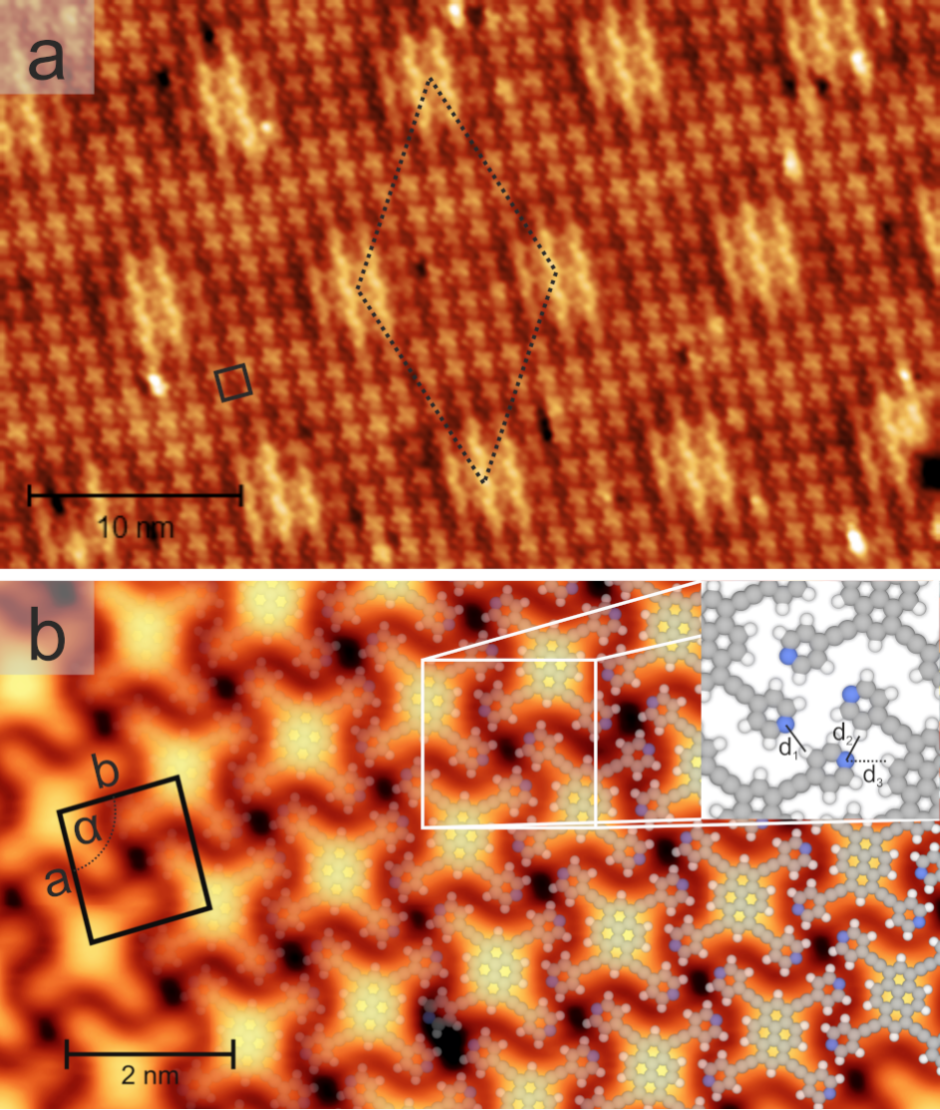
Self-assembly of 1,3,6,8-tetrakis(pyridin-4-ylethynyl)pyrene (**1**, *tetra*) on hBN/Cu(111), as imaged by STM. a) Overview image (2.0 V, 0.1 nA). The moiré pattern of the underlying hBN/Cu(111) caused site-selective gating, as reflected in the hexagonal superlattice in the image (dotted rhombus). b) High-resolution image (0.1 V, 0.2 nA), partially overlaid by structural models. The black tetragon marks the unit cell (lattice vectors *a* and *b*) and the white square highlights the intermolecular interactions (see inset).

Depositing the tetrapyridylpyrene derivate **1** onto *h*BN/Cu(111) at room temperature and subsequent cooling to 6 K gave rise to the formation of extended well-ordered islands even in the submonolayer regime (Figure 2). Imaging these islands at specific sample bias voltages simultaneously showed two patterns with distinct periodicities and symmetries. The rectangular lattice of the X-shaped units corresponded to a densely-packed molecular array (black tetragon in Figure 2), with every unit representing one molecule. The quasihexagonal pattern with larger periodicity (dotted rhombus in Figure 2a) reflected a modulation of the molecular electronic structure imposed by the electronically corrugated *h*BN/Cu(111) support, as discussed in detail below [18,28,37–38]. The high-resolution STM data in Figure 2b gives a closer look at the intramolecular features. Along with the pyrene core, imaged as a large, elongated protrusion, four additional lobes contributed to the X-shaped appearance of the molecular units of **1**. Each of these peripheral protrusions was attributed to one pyridin-4-ylethynyl substituent. Atomistic models (see overlays in Figure 2b), reflecting the gas-phase optimized molecular structure, matched the submolecular contrast in the STM images, and thus supported this assignment. Based on this comparison, we ruled out strong adsorption-induced deformations of the molecule and concluded that the pyrene core adsorbed mostly flat, i.e., the π-conjugated core aligned parallel to the *h*BN sheet. This assembly could be described by a nearly rectangular unit cell including one molecule (see black tetragon in Figure 2b, *a_tetra_* = 1.63 nm ± 0.05 nm, *b_tetra_* = 1.50 nm ± 0.05 nm, and *α_tetra_* = 92° ± 2°). Accordingly, the surface molecular density ρ_tetra/_*_h_*_BN_ of molecule **1** amounted to 0.41 molecules/nm^2^. The molecules interdigitated in both the *a_tetra_* and *b_tetra_* directions, with two distinct interdigitation arrangements, inducing organizational chirality of the achiral pyrene units [48]. Based on the model of the assembly depicted in Figure 2b, the array was stabilized by intermolecular noncovalent interactions, including N^…^H bonds. Distinct organizational motifs could be discriminated (see inset of Figure 2b). The terminal N atom of one molecule could either establish H-bonding with a hydrogen atom of an adjacent pyridyl moiety of a neighboring molecule (link labeled *d_1_* in Figure 2b, projected N^…^H bond length 0.29 nm ± 0.05 nm) or was positioned in the hydrophobic pocket between the pyridyl group and the pyrene core of an adjacent molecule (see d_2_ and d_3_).

#### *trans*-like-disubstituted pyrene

**Figure 3:**
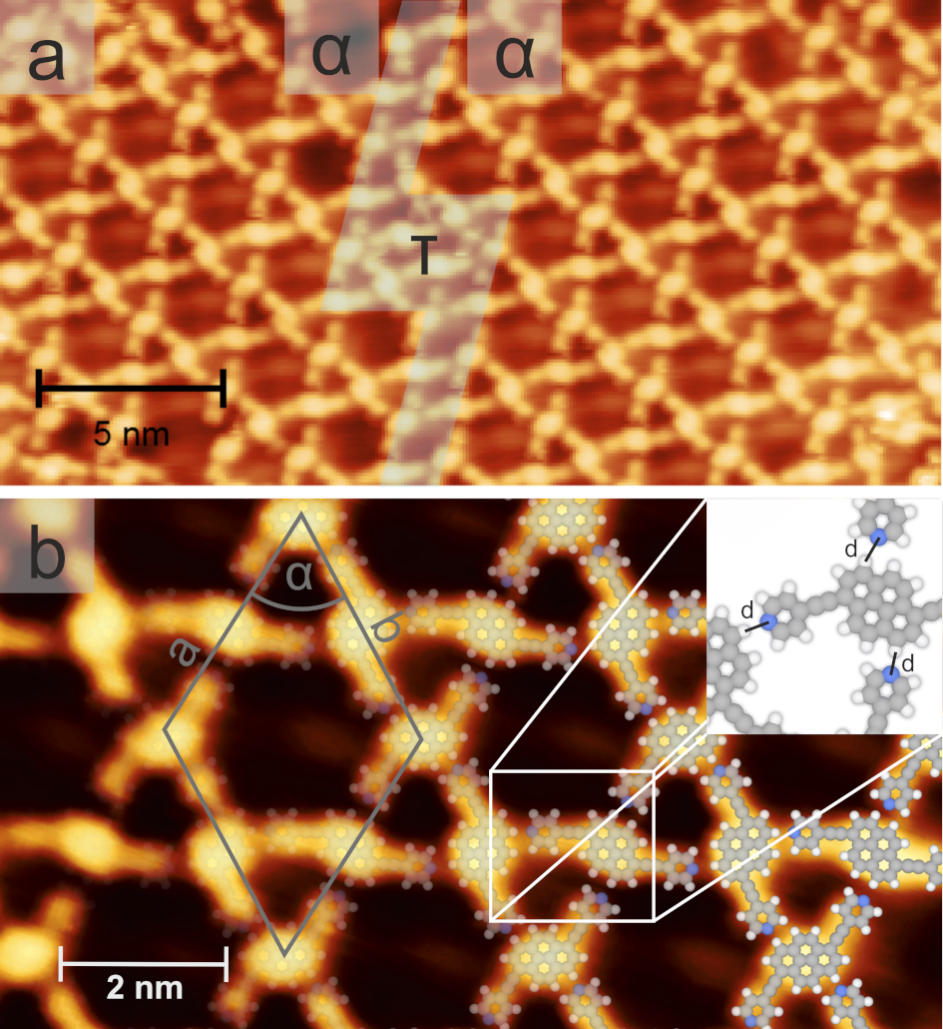
Self-assembly of 1,6-bis(pyridin-4-ylethynyl)pyrene (**2**, *trans*-like) on *h*BN/Cu(111). a) Overview image (1.0 V, 0.1 nA). Two homochiral domains (α) with an open porous kagome structure are connected by a narrow transition region (τ). b) High-resolution STM image (1.0 V, 0.1 nA), partially overlaid by structural models. The grey rhombus shows the unit cell and the white square highlights the intermolecular interactions, represented by the distances *d* in the inset.

Figure 3 shows the STM images of the surface after the deposition of the *trans*-like-disubstituted pyrene derivative **2** on *h*BN/Cu(111). The molecules form open porous networks, featuring a kagome lattice architecture with cavities of two distinct sizes and shapes [42,48,75–76]. At the bias voltage used in Figure 3, the substrate-induced contrast modulation (see below Figure 5c) was not observed. The pyrene **2**, being prochiral, becomes chiral upon surface adsorption [61,77–78], and two stereoisomers can equally be formed on the surface. Each enantiomer segregates into homochiral domains (α and α‘, see Figure 3 and Figure S6, Supporting Information File 1). The lateral extension of the regular, defect-free arrays is usually rather limited (<30 nm), with the narrow transition regions τ representing dislocations (Figure 3a) or mirror domain boundaries (Figure S6, Supporting Information File 1). STM images resolving individual molecules (Figure 3b) revealed a bright protrusion, attributed to the pyrene core, centered between two peripheral lobes, assigned to the pyridin-4-ylethynyl substituents (see structural models in Figure 3b). The unit cell of the kagome lattice is visualized by the grey rhombus in Figure 3b (*a*_trans_ = *b*_trans_ = 3.0 nm ± 0.07 nm, α_trans_ = 60° ± 2°) and contains three molecules. The molecular surface density amounted to about 0.38 molecules/nm^2^, and thus was lower than that of the derivative **1**. The formation of this kagome network was mediated by the formation of intermolecular H-bonds between the pyridyl ligands and the adjacent pyrene cores, with a projected N···H bond length of 0.21 nm ± 0.05 nm (labeled as *d* in Figure 3b). For each chirality, six distinct rotational orientations of the *trans*-like molecule **2**, separated by 30°, were observed on *h*BN/Cu(111) (not shown). Although rare, coexisting densely packed arrays could be detected near the step edges (see Figure S7, Supporting Information File 1).

#### *cis*-like-disubstituted pyrene

**Figure 4:**
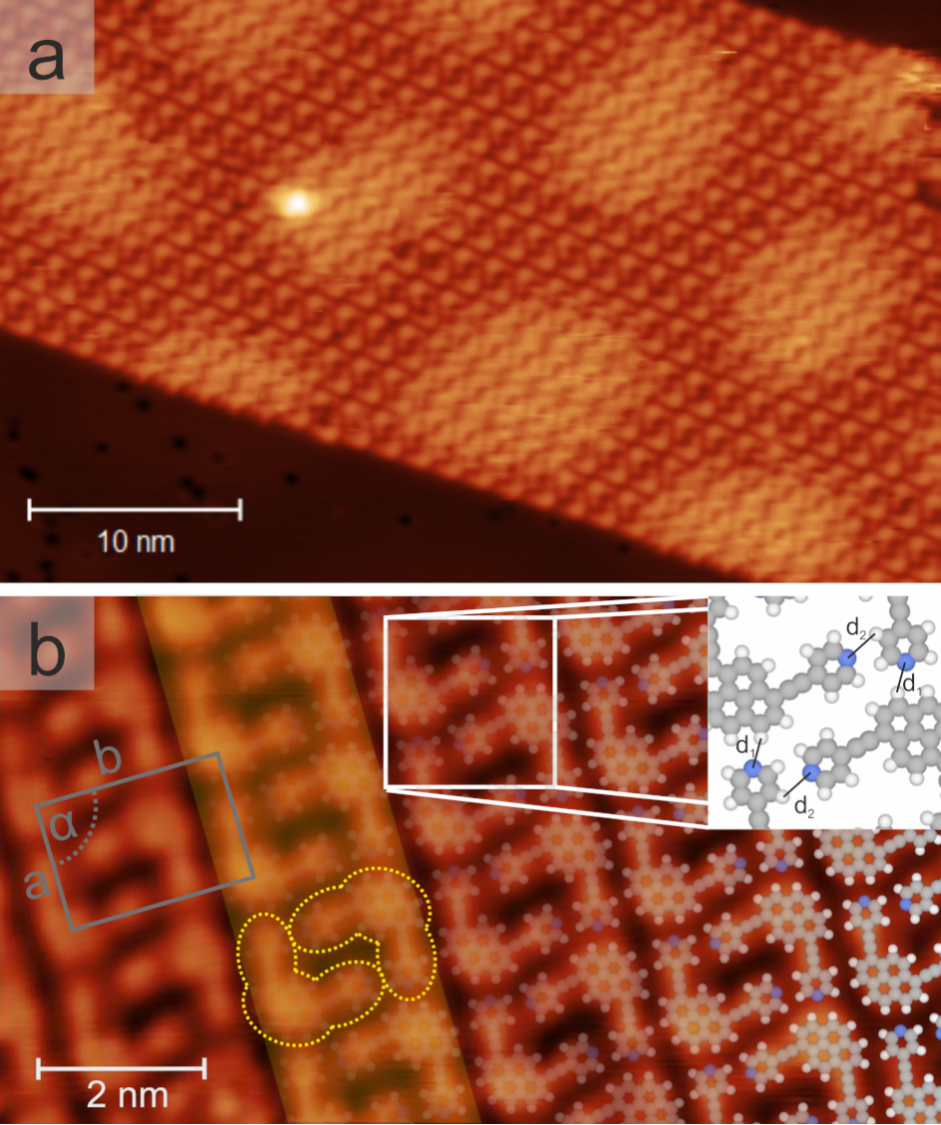
Self-assembly of 1,8-bis(pyridin-4-ylethynyl)pyrene (**3**, *cis-*like) on hBN/Cu(111). a) Overview image (1.38 V, 0.024 nA). Regular islands were formed after rt deposition. Several rows, each containing molecules in two opposite orientations, formed a ribbon-like assembly. The hexagonal moiré pattern of the underlying hBN/Cu(111) was visualized by site-selective gating. b) High-resolution image (1.0 V, 0.27 nA) partially overlaid with the corresponding structural models. The grey rectangle shows the unit cell and the white square highlights the intermolecular bonding of the substituents, schematically represented in the inset. A dimeric motif is marked by the yellow outline, with a dimer row shaded in yellow.

The deposition of the *cis*-like-disubstituted pyrene derivative **3** on *h*BN/Cu(111) at submonolayer coverage yielded extended, densely packed islands featuring straight edges. Figure 4a shows an STM image recorded at a bias voltage where both the molecular lattice and the substrate-induced long-range modulation of the electronic structure are resolved (compare to Figure 2a). High-resolution data (Figure 4b) allowed us to discern individual molecules with submolecular features. Each molecule was characterized by three protrusions, which corresponded to the central pyrene and the two peripheral pyridin-4-ylethynyl substituents, respectively. Two oppositely oriented molecules formed an interdigitated dimeric motif (highlighted by the yellow outlines in Figure 4b) that involved N···H interactions between the pyridyl moieties (*d_2_* = 0.30 nm ± 0.05 nm, inset of Figure 4b). These dimers formed rows, where one N atom of each unit was oriented towards the pyrene core of a neighboring dimer. This noncovalent interaction was described by the distance *d_1_* (0.28 ± 0.05 nm, inset of Figure 4b). Multiple rows, aligned in parallel, with a well-defined registry, constituted the extended islands. As no pyridinic N atom was directly involved in the interrow interaction, the stabilization of the islands was attributed to van der Waals forces. The straight edges reflected the row structure of the islands (see Figure 4a). The unit cell of the assembly (grey rectangle in Figure 4b, *a_cis_* = 2.27 nm ± 0.07 nm, *b_cis_* = 1.57 nm ± 0.05 nm, *α_cis_* = 90° ± 2°) contained two molecules, resulting in a molecular surface density of 0.56 molecules/nm^2^. The dimer of species **3** occured in two interdigitation arrangements [48], giving rise to mirror domains, reflecting organizational chirality. Overall, six distinct rotational orientations of the *cis*-like pyrene molecule **3**, separated by 60°, were observed on *h*BN/Cu(111) (not shown).

#### Binary assemblies

The combination of different pyrene derivatives opens pathways to distinct multicomponent assemblies on *h*BN/Cu(111). For example, the sequential deposition of the *cis*-like derivative **3** and the *trans*-like pyrene **2** afforded binary architectures, including regular densely packed arrays and kagome networks hosting the species **3** in the large cavities (Figure S8, Supporting Information File 1).

### Scanning tunneling spectroscopic measurements

**Figure 5:**
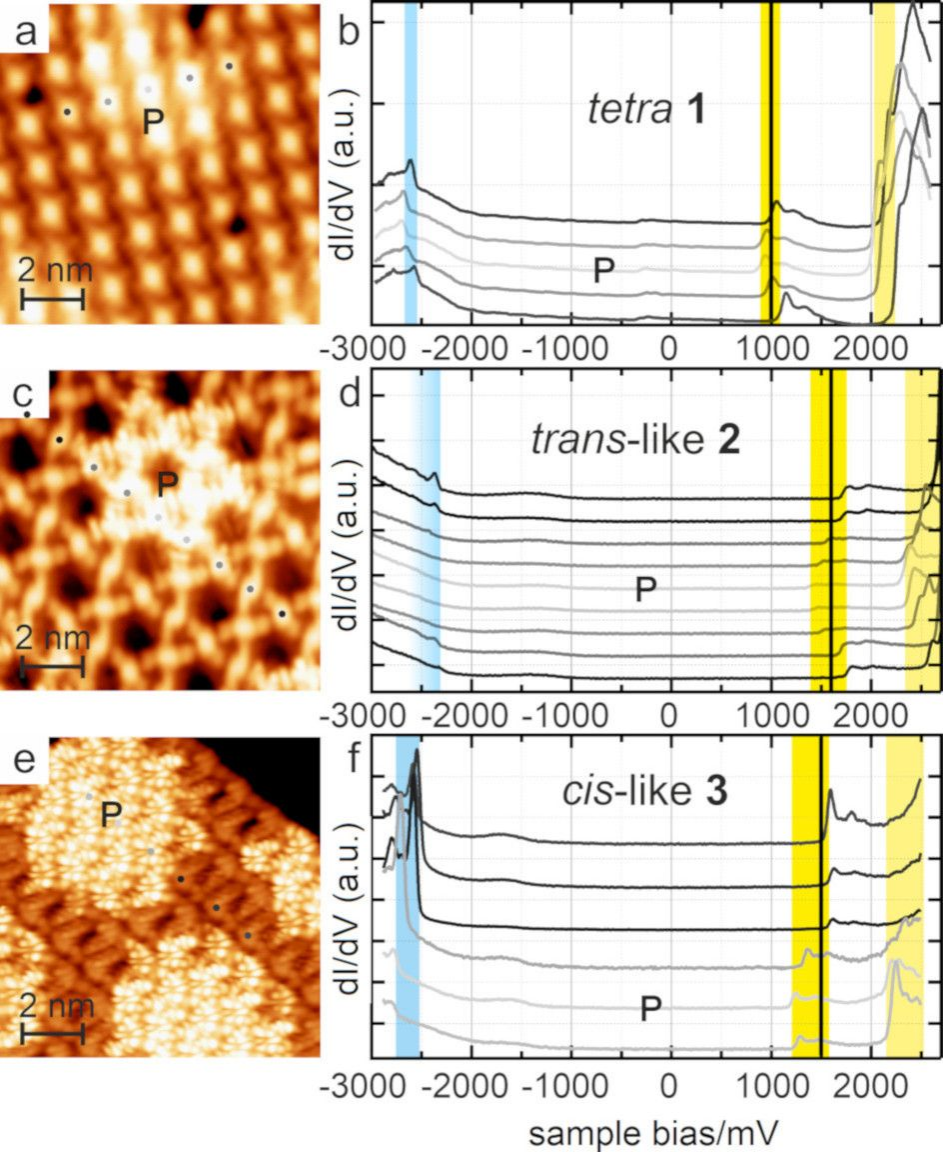
dI/dV signatures of the pyrenes **1**–**3** on *h*BN/Cu(111) and template-induced gating. a), c), and e) STM images of the *tetra*, *trans*-like, and *cis*-like pyrenes on *h*BN, taken at a sample bias at the onset of an unoccupied MO (a: 1 V, 0.04 nA; c: 1.6 V, 0.2 nA; and e: 1.5 V, 0.1 nA). The bright areas represent the pore regions of the *h*BN/Cu(111) support. b), d), and f) dI/dV spectra recorded on the center of molecules at the position of the grey dots in a), c), and e). The darker the color of the dot and the corresponding spectrum, the larger the lateral distance to the *h*BN pore. HOMO, LUMO, and LUMO+1 are indicated by blue, yellow, and light yellow boxes, respectively. Black vertical lines reflect the sample bias voltage of the corresponding STM image (tip stabilization parameters–b: 0.8 V, 0.25 nA; d: 2.7 V, 0.2 nA; and f: 2.5 V, 0.2 nA).

Next, the electronic structure of the functionalized pyrene derivatives **1–3** on *h*BN/Cu(111) was addressed. Specifically, we performed bias-dependent STM imaging and dI/dV spectroscopy to probe the influence of the substitution, in conjunction with the distinct assemblies, on the molecular electronic states. Additionally, the role of the electronic landscape of the *h*BN/Cu(111) support, inducing a periodic modulation of the pyrene electronic structure via site-selective gating, is highlighted. Figure 5 shows a series of dI/dV spectra recorded above the molecular centers of the pyrene derivative **1** (Figure 5b), **2** (Figure 5d), and **3** (Figure 5f) at different adsorption positions on *h*BN/Cu(111), as indicated by grey markers in the corresponding STM images in Figure 5a, 5c, and 5e, respectively. The spectra of all three compounds revealed well-defined features in the occupied (negative sample bias) and unoccupied (positive sample bias) spectral regions. Tentatively, the characteristic signatures were assigned to the HOMO, LUMO, and LUMO+1.

The colored bars in Figure 5 highlight the energy positions of these frontier orbitals (determined as the bias voltage at the half maximum of the MO leading edge). The observation of well-defined, narrow molecular resonances and large HOMO–LUMO gaps evidenced a reduction of the electronic molecule–support interactions by the *h*BN spacer layer, as previously reported for adsorbates on *h*BN/Cu(111) [28,35–38] and other *h*BN/metal supports [18–20,79–80]. The dI/dV modulations observed within the gap were attributed to the *h*BN/Cu(111) support and tip states, respectively (vide infra).

A closer look at the dI/dV spectra in Figure 5b, 5d, and 5f revealed a site-dependent shift of the molecular resonances (MOs), an effect attributed to site-selective gating by the underlying *h*BN/Cu(111) support. This template featured a moiré pattern with areas of low local work function (pores, P) and high local work function (wires, W) [25,29–30], which was reflected by the molecular level alignment, as measured by dI/dV spectroscopy [28,35–37]. On pore areas, the MOs were shifted downwards (i.e., towards the Fermi level (*E*_F_) for unoccupied states, and away from *E*_F_ for occupied states) compared to the wire areas. The grayscale of the spectra in Figure 5 reflect the proximity to a pore, with light grey indicating adsorption on pore areas and dark grey reflecting adsorption on (or near) wire areas. Accordingly, the periodic modulation of the pyrene electronic structure induced by the moiré pattern of the underlying *h*BN/Cu(111) could directly be visualized in STM images recorded at suitable sample bias voltages (see Figure 2a, Figure 4a and Figure 5a, 5c, 5d, as well as Figures S9 and S10, and the movie in Supporting Information File 2). At bias voltages (see vertical black lines in Figure 5b, 5d, and 5f) where a specific MO could only contribute to the tunneling current in the pore areas, a contrast between the pore and the wire areas emerged in the STM images, with the molecules on the pores featuring an increased apparent height and a modified submolecular contrast (vide infra).

**Figure 6:**
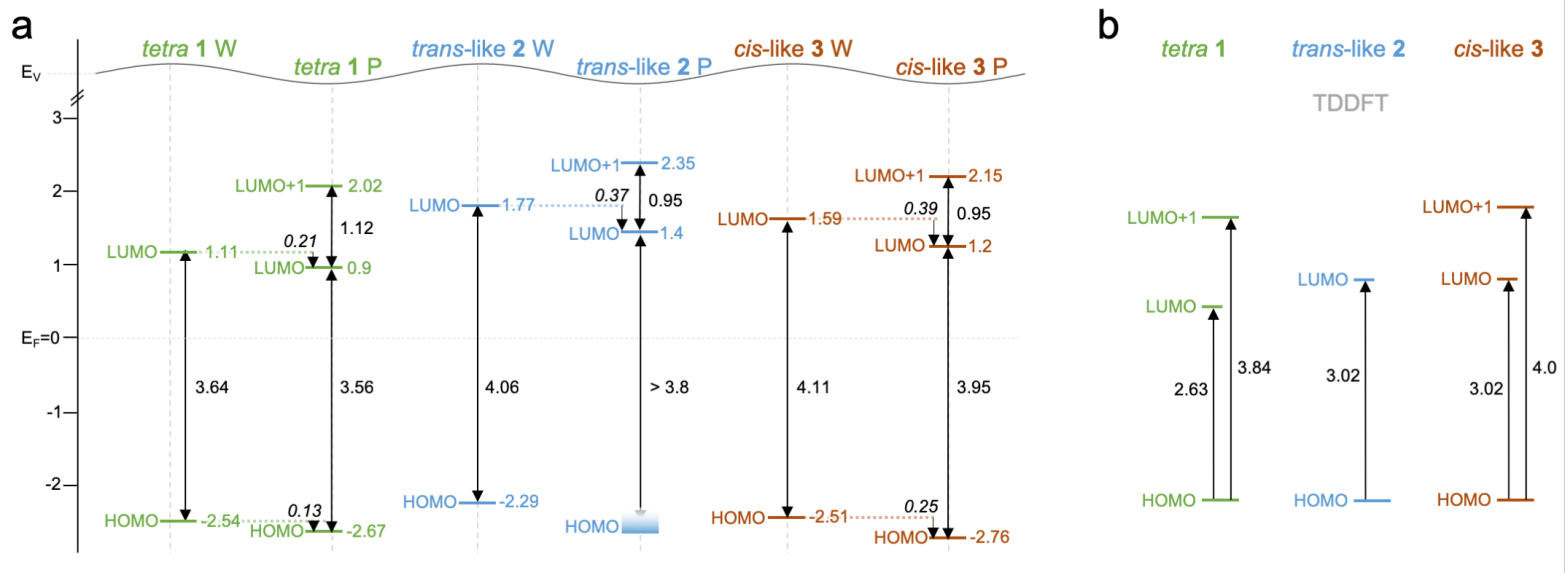
Diagrams summarizing the spectroscopic values determined for the pyrene derivatives **1**–**3**. a) STS data for *h*BN/Cu(111), sketching the energy level alignment (compare to Figure S11, Supporting Information File 1). b) Time-dependent DFT (TDDFT)-calculated excitation energy in explicit solvent reflecting the UV–vis measurements in toluene solution (see Tables S1–S3). The units are in Volt. P: pore area, W: wire area.

Next, the dI/dV signature of the tetrasubstituted pyrene derivative **1** (Figure 5b) was in the focus. A site-dependent shift was evident for all MOs: The LUMO energy ranged from about 0.89 V (P) to 1.11 V (W), revealing a shift of ≈0.22 V, as highlighted by the width of the yellow box in Figure 5b. A similar shift (≈0.23 V) was observed for the LUMO+1. The HOMOs were observed at voltages between −2.54 V (W) and −2.66 V (P, width of the blue box ≈ 0.13 V). Thus, for the species **1**, the spectra revealed STM-derived HOMO–LUMO gaps of 3.64 eV (in the pore areas) and 3.56 eV (in the wire areas). The wire spectrum for the molecule **1** was recorded at a larger distance from the real wire position, as compared to **2** and **3** (see Figure 5a, 5c, and 5e). The voltage-dependent STM contrast and the site-dependent variation of spectral features described above for the derivative **1** was analogously observed for the derivatives **2** and **3** (see blue and yellow boxes in the Figure 5(d and f). However, the MOs occurred at energies characteristic for the three different pyrene species, yielding distinct STS gaps, as summarized in Figure 6a.

**Figure 7:**
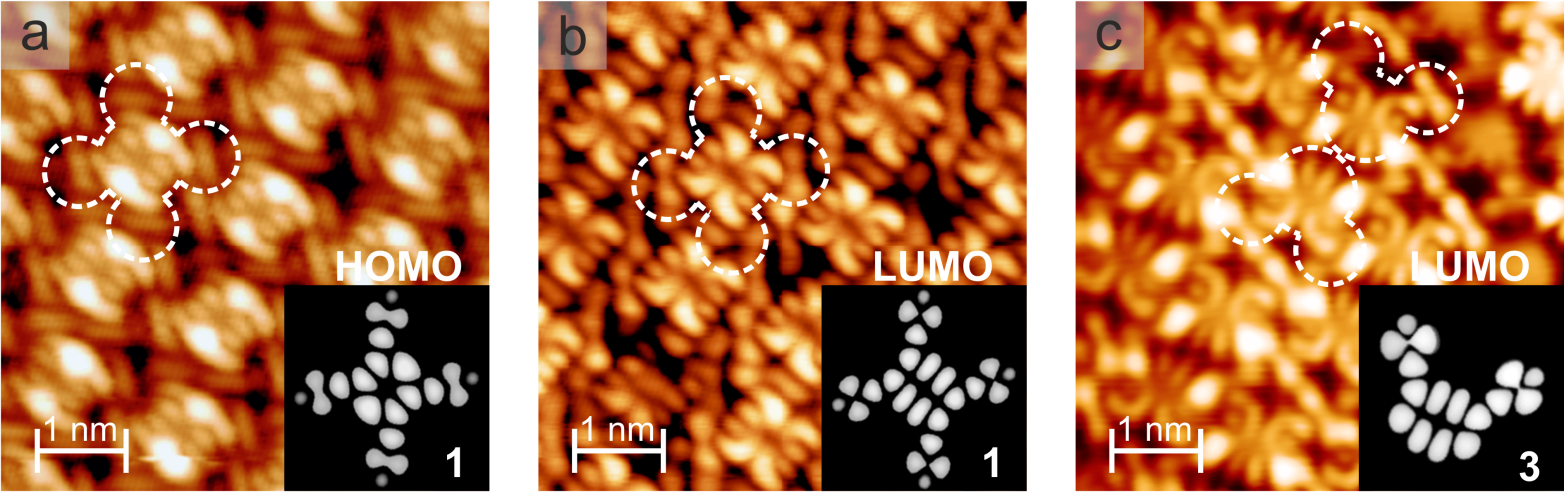
High-resolution STM images revealing the bias-dependent intramolecular contrast of the pyrene derivatives **1** and **3** on *h*BN/Cu(111), recorded with a tip of unknown termination. a) Array of the tetrasubstituted species **1** imaged at a bias voltage of –2.87 V (0.06 nA) and b) at 1.4 V (0.04 nA). c) Assembly of the *cis*-like derivative **3**, imaged at 1.5 V (0.27 nA). The dashed contours highlight the individual molecules. The insets show the EHT-calculated MOs corresponding to the HOMO (a) and the LUMO (b and c) of the free molecule (compare to Figure 2).

Figure 7 shows the high-resolution STM images of the pyrene derivatives **1** and **3** recorded at bias voltages where the MOs were accessible. In contrast to imaging at bias voltages within the HOMO–LUMO gap, where the STM images essentially reflect the molecular shape (Figure 2b, Figure 3b, and Figure 4b) [17], distinct intramolecular features of electronic origin emerged (see also Figure S12, Supporting Information File 1). A comparison to the Kohn–Sham orbitals (see Figure 1) and to the frontier orbitals calculated in the EHT scheme (see insets in Figure 7) revealed striking similarities. For example, the number of antinodes as well as the nodal planes between the experimental images and the calculations were in agreement, strongly suggesting that the STM contrast indeed reflected the MO contributions, corroborating the assignment of spectral features to the HOMOs and the LUMOs. Furthermore, the agreement between the gas-phase calculations and STM data indicated that no charging occurred upon pyrene adsorption on *h*BN/Cu(111) [37].

**Figure 8:**
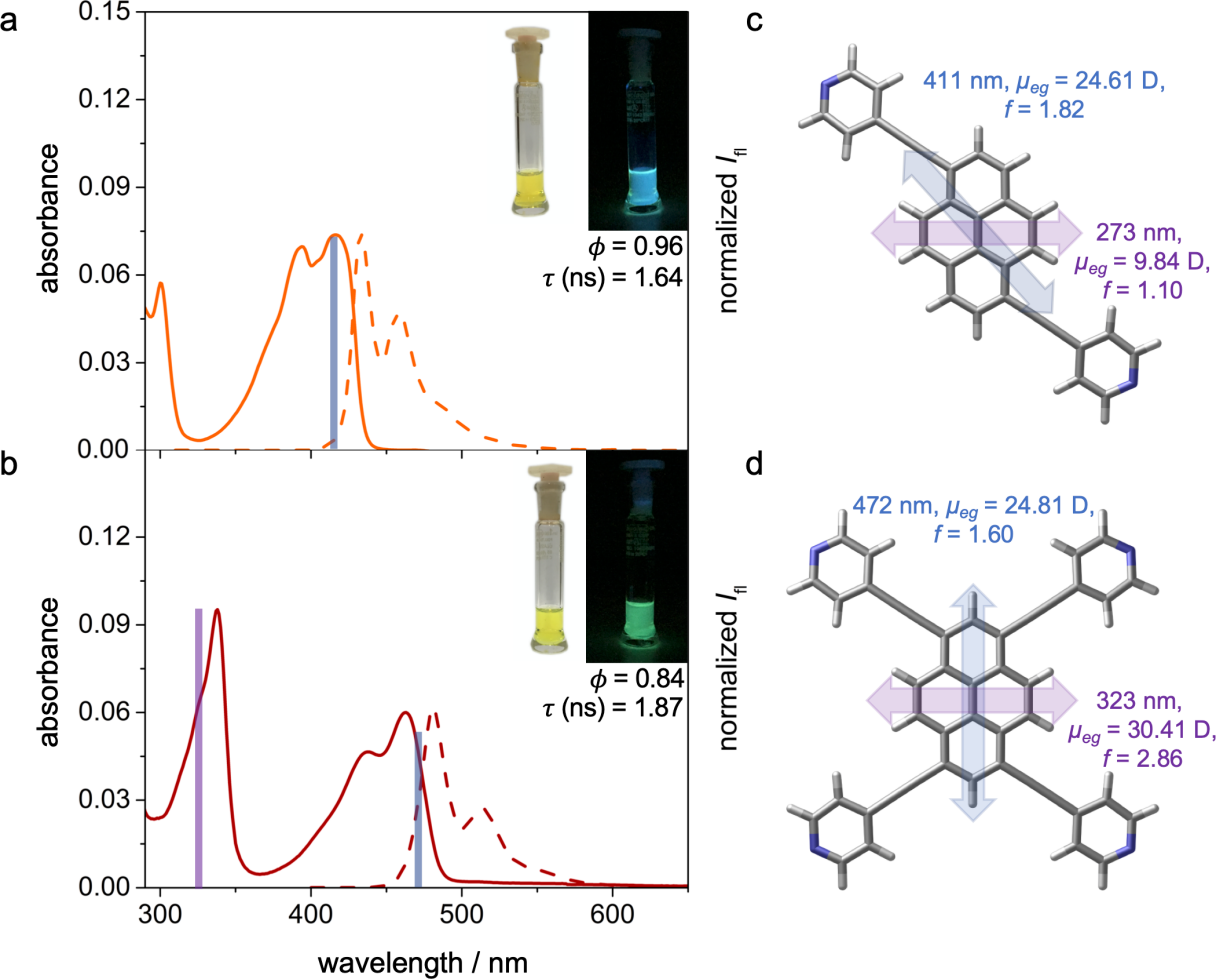
Optical characterization. a) and b) UV–vis absorption (solid line) and emission spectra (dotted line) of the *trans*-like (a, orange line) and tetrasubstituted (b, red line) pyrenyl derivatives in toluene (*c* = 1.0 × 10^−6^ M) at rt. Insets: photographs of toluene solutions in daylight and upon irradiation with a hand-held UV lamp (λ_ex_ = 365 nm). c) and d) Calculated transitions (blue and purple lines), transition dipole moments (μ_eg_), and oscillator strengths (*f*), as determined by TDDFT (CAM-B3LYP, 6-31G**, explicit solvent: toluene).

To complement the on-surface investigations, we measured the optical gap by means of UV–vis spectroscopic characterization in solution (toluene) of the *trans*-like and tetrasubstituted pyrenyl derivatives (Figure 8a and Figure 8b). The *cis*-substituted pyrene **3** showed a lower solubility in solvents such as toluene, and therefore the optical properties could not be measured, although (TD)DFT calculations indicated a similarity to the *trans*-like isomer (Figure S3 and Tables S1–S3, Supporting Information File 1). The absorption spectrum of the *trans*-substituted pyrene **2** showed two main absorption peaks centered at 416 and 394 nm (2.98 and 3.15 eV, respectively), with another lower-absorption transition at a higher energy (300 nm, 4.13 eV). The selective excitation of the low-energy absorption peak lead to a strong emission at 433 and 458 nm (2.86 and 2.71 eV), with a quantum yield of 96% (determined using coumarin 153 in an EtOH solution as a reference). As expected, the tetrasubstituted pyrene **1** showed electronic transitions that were bathochromically shifted to higher wavelengths, with two main bands observed at 463 and 438 nm (2.68 and 2.83 eV) and one at a higher energy at 338 nm (3.67 eV). The excitation of the lowest-energy bands also led to a strong emissive band, centered at 481 and 513 nm (2.58 and 2.42 eV), with a quantum yield of 84%. The same optical transitions seemed to be involved in both the absorption and emission processes, as confirmed by a mirror symmetry of the fluorescent spectral envelope compared to the lowest-energy absorption transitions. Moreover, the small Stokes shifts (17 and 18 nm for the *trans*- and tetrasubstituted pyrene derivatives, respectively) confirmed the fact that for both structures, the ground and excited states were similar, while the matching excitation and absorption spectra pointed at an efficient radiative deactivation of the excited state (Figure S13, Supporting Information File 1) [71].

TDDFT calculations (CAM-B3LYP/6-31G**, toluene CPCM solvation) [69,73] were further performed, and the results are summarized in Figure 8c, Figure 8d, and Tables S1–S3. The main transitions for the *trans*-pyrene seemed to originate from the HOMO→LUMO transition (estimated at λ = 411 nm (3.02 eV), *f* = 1.82) and the HOMO−1→LUMO/HOMO→LUMO+2 transitions (estimated at λ = 273 nm (4.54 eV), *f* = 1.10), with the transition dipole moments aligned along the 1- and 6-positions (towards the pyridin-4-ylethynyl termini) and the short molecular axes of the central pyrene core. On the other hand, the tetrapyrenyl transition dipole moments were aligned along the long and short molecular axes of the pyrene core, with the two main transitions having HOMO→LUMO (estimated at λ = 472 nm (2.63 eV), *f* = 1.60) and HOMO−1→LUMO/HOMO→LUMO+1 contributions (estimated at λ = 323 nm (3.84 eV), *f* = 2.84).

## Discussion

### Self-assembly

In this section, we will put forward a comparative discussion of the self-assembly of pyridin-4-ylethynyl-functionalized pyrenes on *h*BN/Cu(111) and on Ag(111) [48]. The molecular density of tetrapyridylpyrene **1** in arrays on *h*BN/Cu(111) was higher compared to Ag(111) (ρ_tetra/_*_h_*_BN_ = 0.41 molecules/nm^2^ vs ρ_tetra/Ag_ = 0.37 molecules/nm^2^). Accordingly, distinct differences were observed in the unit cell dimensions and the intermolecular alignment, even though the N···H interactions contributed to the self-assembly in both cases. At the same time, the intermolecular distance along the unit cell vector *b* was similar on *h*BN/Cu(111) and Ag(111), the separation along the unit cell vector *a* was clearly reduced on *h*BN, reflecting the minimum energy interdigitation configuration, determined by basic molecular mechanics modeling for two modules of **1** in the gas phase. Thus, we tentatively assigned the higher packing density on *h*BN to the reduced site-specific molecule–support interactions. Indeed, distinct registries of the derivative **1** on the Ag(111) atomic lattice were reported [48]. However, additional effects potentially perturbing the intermolecular alignment, such as subtle differences in the pyridine tilt angle (out of the surface plane), could not be excluded based on our experimental observations. The kagome-like porous network architectures formed by the *trans*-like-disubstituted pyrene derivative **2** on *h*BN/Cu(111) and Ag(111) matched within the experimental precision. Accordingly, in contrast to the derivative **1**, no effect of a reduced site-specific molecule–support interaction was discernible for the derivative **2**. Thus, we speculate that the unit cell of an unsupported supramolecular structure, being close to the one observed on *h*BN, can be accommodated on the Ag(111) atomic lattice, where a commensurate structure was reported [48].

The assembly of densely packed islands of the *cis*-like-disubstituted pyrene derivative **3** on *h*BN/Cu(111) at a low coverage was in stark contrast to the formation of extended chains and disordered two-dimensional agglomerates on Ag(111) [48]. The prominent head-to-head coupling motif on Ag(111) was not observed on *h*BN. This corroborated previous assumptions about interactions of the pyridyl termini with Ag surface atoms mediating the chain formation [48]. The interdigitated dimeric motif and the row-like island structure observed on *h*BN/Cu(111), however, matched the findings in the monolayer regime on Ag(111). Accordingly, as for the *trans*-like pyrene **2**, the support played a minor role in the intralayer structure of the extended molecular arrays.

Nonetheless, the results for the pyrenes **1**–**3** on *h*BN/Cu(111) clearly demonstrated that the support did affect the rotational alignment of molecules and assemblies. As discussed for the *cis*- and *trans*-like isomers, the molecules were oriented along specific directions, separated by 60 or 30°, respectively. This suggested that the ethynyl-functionalized pyrene cores were aligned with the high-symmetry directions of the *h*BN/Cu(111) support, and thus yielded only distinct orientations of the supramolecular architectures. Indeed, DFT calculations have predicted the adsorption of the pyrene with the aromatic rings centered above the N-positions of a free-standing *h*BN sheet [81]. Preferred alignments have also been reported for low coverages of PTCDA and MnPc on the strongly corrugated *h*BN/Rh(111) support [21–22,80], with computational modeling showing PTCDA rings positioned above the N sites of a *h*BN flake [14]. In contrast, no preferred orientations have been identified for a hydrocarbon lander molecule (i.e., DBP) on *h*BN/Pt(111) [19].

The formation of extended domains for the pyrene derivatives **1**–**3** on *h*BN/Cu(111) even at a submonolayer coverage was reminiscent of findings for carbonitrile-functionalized porphyrins [38] and quaterphenylenes [36] but contrasted the site-selective adsorption on pore areas reported for TCNQ, porphines, and fluorinated phthalocyanines [28,33,37]. Taken all together, these observations demonstrated that distinct tectons with peripheral recognition moieties can afford the assembly of extended supramolecular architectures also on electronically superstructured *h*BN platforms.

### Spectroscopic investigations and determination of the molecular gap

Both the STS studies addressing the adsorbed species on the single-molecule level and the optical spectroscopy in solution confirmed the gap reduction upon increasing the number of pyridin-4-ylethynyl substituents, as predicted by DFT calculations (Figure 6). Within the experimental uncertainty, the STM-derived HOMO–LUMO gap of the disubstituted species **2** and **3** was the same. Interestingly, however, the spectra of the *cis*-like derivative showed a rigid energy shift of ≈0.2 V for all MOs (the LUMO was shifted towards *E*_F_ and the HOMO away from *E*_F_), compared to the *trans*-like species. Such a rigid shift can be caused by charges in the molecular layer (biasing nearby molecules) and by local modifications of the work function affecting the interfacial level alignment [9,35,82–83]. As the STM/STS experiments provided no indication of charging, we ruled out a dominating contribution of the former effect. Instead, we tentatively assigned the rigid MO shift to the differences in the self-assembled structures, namely a densely packed arrangement for the derivative **3** and the porous structure for the derivative **2**. With the *h*BN/Cu(111) work function being modified upon molecular adsorption (Figure S11, Supporting Information File 1), open-porous structures would feature a smaller work function shift compared to densely packed molecular films. In the absence of charge transfer, the work function was assumed to decrease upon the adsorption of pyrenes on *h*BN/Cu(111) [84]. Accordingly, the derivate **3** would experience a lower average local work function than the derivate **2**, resulting in a downward shift of the MOs, as observed in the experiment (Figure 5 and Figure 6). Indeed, the molecular packing effect was corroborated by comparing the MO signatures of the molecule **2** in the porous and densely packed assemblies, respectively. In the latter, the MOs were centered at lower energies, similar to those observed for the densely packed islands of the species **3**. The HOMO–LUMO gap (≈4.1 V) was not considerably affected by the different molecular organization (see Figure S7d, Supporting Information File 1). In contrast to recent reports on F_16_CoPc/*h*BN/Cu(111) [37] and PTCDA/NaCl/Ag(111) [83], an additional effect of the molecular aggregation and packing density, namely the screening by neighboring molecules, yielding a reduction of the STS-derived HOMO–LUMO gap, did not seem to play a dominant role here. The packing might have also influenced the adsorption geometry (registry, subtle conformational adaption) without manifestation in the experiments.

For all three pyrene derivatives, the calculated energy difference between the HOMO and the LUMO (Figure 1 and Figure S2, Supporting Information File 1) was considerably smaller than the one measured by STS (Figure 6a). This discrepancy was tentatively assigned to an underestimation of the gap by the applied DFT scheme. The energy difference between the calculated ionization potential (IP) and the electron affinity (EA) on the other hand exceeded the STS gap. The STS-derived separation between the LUMO and the LUMO+1 was reduced for the disubstituted species compared to the tetrasubstituted pyrene **1** (see Figure 6). This trend matched the DFT prediction (Figure 1) and was assigned to the stabilization of the LUMO for tetrasubstitution.

The template-induced gating by the electronic landscape of the *h*BN/Cu(111) similarly affected all three pyrene derivatives. The energy shift of the LUMO between the pore and wire areas (0.21 V for **1**, 0. 37 V for **2**, and 0.39 V for **3**, see Figure 6) agree well with the previously reported MO shifts on *h*BN/Cu(111) (ranging from 0.25 to 0.4 V [28,35–37]) and reflect the local work function variation across the moiré pattern [25,29–30]. In a recent study on F_16_CoPc/*h*BN/Cu(111), we demonstrated that the local work function difference between the pore and wire regions was preserved upon molecular adsorption [37]. Note, that the smaller shift for the molecule **1** was attributed to the larger distance of the respective wire spectrum from the real wire position (vide supra). The shrinking of the HOMO–LUMO gap in the pore areas, consistently observed for the species **1**–**3**, was attributed to the increased molecule–support interactions in the pores [28], where the *h*BN was located closer to the Cu(111) support [29]. Accordingly, no rigid shift of the occupied and unoccupied molecular electronic states occurred, as reflected in the HOMO energy shifts (e.g., 0.25 V for **3**), falling below the LUMO shifts (see Figure 6). Dissimilar responses of distinct MOs to work function variations were previously discussed, e.g., for pentacene on dielectric decoupling layers [23].

The assignment of the dI/dV signature to the MOs was corroborated by resolving the submolecular features in high-resolution STM images at these bias voltages, reflecting the frontier orbitals of the free pyrenes (Figure 7 and Figure S12, Supporting Information File 1). Nonetheless, with additional features observed in the gap of the dI/dV spectra (see Figure 5b, 5d, and 5f), the unambiguity of such an identification needs to be addressed. The step-like increase in the dI/dV signal at ≈−350 meV (Figure 5b) reflected the electronic interface state of *h*BN/Cu(111) [25]. The support also accounted for the steadily increasing background contribution at negative bias voltages exceeding −2V (apparent in all spectra) [28,31,41].

A comparison between the values of the HOMO–LUMO gaps and the excitation energy obtained by STS and UV–vis absorption (see Figure 6) revealed that the optical gap seemed to be smaller than the electronic gap. This was expected due to the intrinsic differences in the measurement process of these gaps [85]. However, studies comparing orbital-resolved STM/STS data to optical gaps are scarce [86]. In addition to this effect, the adsorption and supramolecular organization on *h*BN could sensitively affect the optical transitions [14,16,21]. For example, the fluorescence peak energy of perylene derivatives was reduced considerably (0.3–0.4 eV) upon the adsorption on bulk-like *h*BN [14]. Accordingly, the gaps and excitation energy compiled in Figure 6 did not allow for a direct comparative assessment of the interaction of the pyrene derivatives with the environment on hBN or in solution.

## Conclusion

In summary, we characterized the electronic and photophysical properties as well as the self-assembly abilities of a family of functionalized pyrenes bearing four and two pyridin-4-ylethynyl substituents. Specifically, UV–vis spectroscopic investigations in toluene solution and the dI/dV measurements at the vacuum/solid interface showed that the electronic and optical gaps could be engineered by the number of substituents. This agrees with the DFT computations predicting a gap reduction when proceeding from unsubstituted pyrene cores to di- and tetrasubstituted derivatives. Applying an atomically thin *h*BN sheet as decoupling layer, the electronic structure of the pyrene derivatives was probed at the submolecular level, visualizing an MO-like contrast and evidencing effects of supramolecular organization. Importantly, three distinct molecular resonances could be detected, and the response to template-induced gating revealed weak molecule–support interactions. The STM data showed an electronic patterning of the pyrene films with periodicities in the single digit nanometer range. Furthermore, we provide a first case study for the self-assembly of advanced porous structures on *h*BN monolayers, introducing a chiral kagome-like architecture. These results gave unprecedented information on the spatially and energetically resolved molecular states of a photoactive polycyclic aromatic hydrocarbon on a solid support and highlight the potential to engineer interfacial electronic and optoelectronic properties by molecular design and surface organization.

## Experimental

### STM and STS

All scanning-probe experiments were performed in a custom-designed UHV system hosting a CreaTec low-temperature STM (CreaTec Fischer & Co. GmbH, createc.de) and providing a base pressure below 1 × 10^–9^ mbar. The monocrystalline Cu(111) substrate was cleaned by repeated Ar^+^ sputtering cycles at an energy of 800–1000 eV, followed by annealing at 1070 K. Monolayer *h*BN was grown via chemical vapor deposition using borazine ((HBNH)_3_, Katchem spol s.r.o, www.katchem.cz), following a protocol described previously [25]. Subsequently, a submonolayer coverage of the pyrene modules was deposited by organic molecular beam epitaxy from thoroughly degassed quartz crucibles held at 450–500 K. During deposition, the Cu(111) surface was kept at rt, and the pressure remained below 2 × 10^–9^ mbar. The STM images were acquired in constant current mode, with the sample being held at ≈6 K using electrochemically etched W tips. In the figure captions, voltages refer to the bias voltage applied to the sample. Differential conductance (dI/dV) spectra were recorded using the lock-in technique (*f* = 969 Hz, *V*_rms_= 18 mV). Reducing the tip-sample distance by increasing the current by a factor of five for the pyrene derivative **1** had no significant effect on the measured gap. Very broad spectral features (e.g., the peaks around −1.43 V in Figure 5d and −1.65 V in Figure 5f) that did not shift considerably with the lateral position of the spectrum were attributed to the tip states and not to the pyrene-related molecular resonances.

### Photophysical investigations

The toluene solutions of the *trans*- and tetrasubstituted pyrenes (ACS spectroscopic grade, Sigma-Aldrich) were subjected to sonication and heating cycles, and then left to cool to rt before recording the absorption and emission spectra. Absorption spectra were recorded using air-equilibrated solutions at rt, with an Agilent Cary 5000 UV–vis spectrophotometer using quartz cells with a path length of 1.0 cm. Emission spectra were recorded on an Agilent Cary Eclipse fluorescence spectrofluorometer. Emission lifetime measurements were performed on a JobinYvon-Horiba FluoroHub single-photon-counting module, using nano-LED-pulsed sources at 295 or 372 nm. The quantum yield measurements were performed using the relative determination, with coumarin 153 (C153, ϕ = 0.53 in ethanol) as the standard (st) [87]. The fluorescence quantum yields were then calculated according to Equation 1:

[1] $$\phi_{x}=\phi_{st}\cdot \frac{I_{x}}{I_{st}}\cdot \frac{\eta_{x}^{2}}{\eta_{st}^{2}}$$

Therein, *I* is the measured integrated fluorescence emission intensity, η is the refractive index of the solvent, and ϕ is the quantum yield.

### Computational methods

DFT calculations were performed using the Gaussian 09 (Revision D.01) program package [88]. The starting geometries were obtained from molecular mechanics or semiempirical models, followed by DFT geometry optimizations on unconstrained *C*_1_ symmetry. Geometry optimizations were followed by frequency calculations on the optimized structures, which confirmed the existence of minima. DFT calculations were performed using the hybrid functional B3LYP [89–91], with the 6-31G(d,p) basis set. Electronic transitions (up to 10 states) were calculated by means of TDDFT [92] using the CAM-B3LYP [93] functional in combination with the 6-31G(d,p) basis set [73]. The conductor-like polarizable continuum model (CPCM) was used to introduce nonspecific solvation effects. The spectra were generated either with GaussView 5 [94] or GaussSum [95], assuming a half-width of 0.15 eV for proper simulation. The IP and EA were determined as the vertical energy difference between the neutral molecule and the cation/anionic forms, respectively, following Equation 2 and Equation 3 [96]:

[2] $$IP=E_{cation}-E_{neutral}$$

[3] $$EA=E_{neutral}-E_{anion}$$

The resulting values (in eV) were: tetrasubstituted pyrene **1**: IP = 6.49, EA = 2.14; *trans*-like pyrene **2**: IP = 6.58, EA = 1.50; and *cis*-like pyrene **3**: IP = 6.58, EA = 1.48.

## Supporting Information

File 1 Additional computational results (including electronic transitions, electrostatic potential, Cartesian coordinates of optimized structures) and additional STM data (including bias-dependent imaging series and bicomponent assemblies).

File 2 STM image sequence with increasing bias voltage covering the LUMO and LUMO+1 spectral regions.
