# Supplementary material for: Self-assembly and spectroscopic fingerprints of photoactive pyrenyl tectons on hBN/Cu(111)
Source: Beilstein J Nanotechnol. 2020 Sep 29;11:1470–83. doi: 10.3762/bjnano.11.130 (PMC7537405; doi:10.3762/bjnano.11.130)
Supplement: File 1 — Additional computational results (including electronic transitions, electrostatic potential, Cartesian coordinates of optimized structures) and additional STM data (including bias-dependent imaging series and bicomponent assemblies). [file Beilstein_J_Nanotechnol-11-1470-s001.pdf]

BEILSTEIN JOURNAL OF NANOTECHNOLOGY

Supporting Information

for

Self-assembly and spectroscopic fingerprints of photoactive pyrenyl tectons on *h*BN/Cu(111)

Domenik M. Zimmermann, Knud Seufert, Luka Đorđević, Tobias Hoh, Sushobhan Joshi, Tomas Marangoni, Davide Bonifazi and Willi Auwärter

Beilstein J. Nanotechnol. **2020,** *11,* 1470–1483. doi:10.3762/bjnano.11.130

Additional computational results (including electronic transitions, electrostatic potential, Cartesian coordinates of optimized structures) and additional STM data (including bias-dependent imaging series and bicomponent assemblies)

License and Terms: This is a supporting information file under the terms of the Creative Commons Attribution License (https://creativecommons.org/licenses/by/4.0). Please note that the reuse, redistribution and reproduction in particular requires that the authors and source are credited.
The license is subject to the *Beilstein Journal of Nanotechnology* terms and conditions: (https://www.beilstein-journals.org/bjnano)

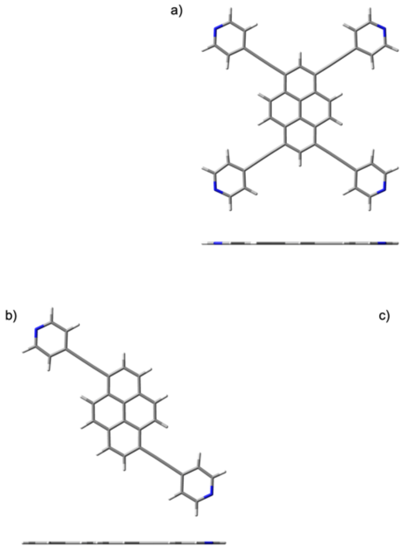

Figure S1. Top and side views of optimized structures at the B3LYP/6-31G (d,p) level of theory for (a) pyrene **1**, (b) **2** and (c) **3**.

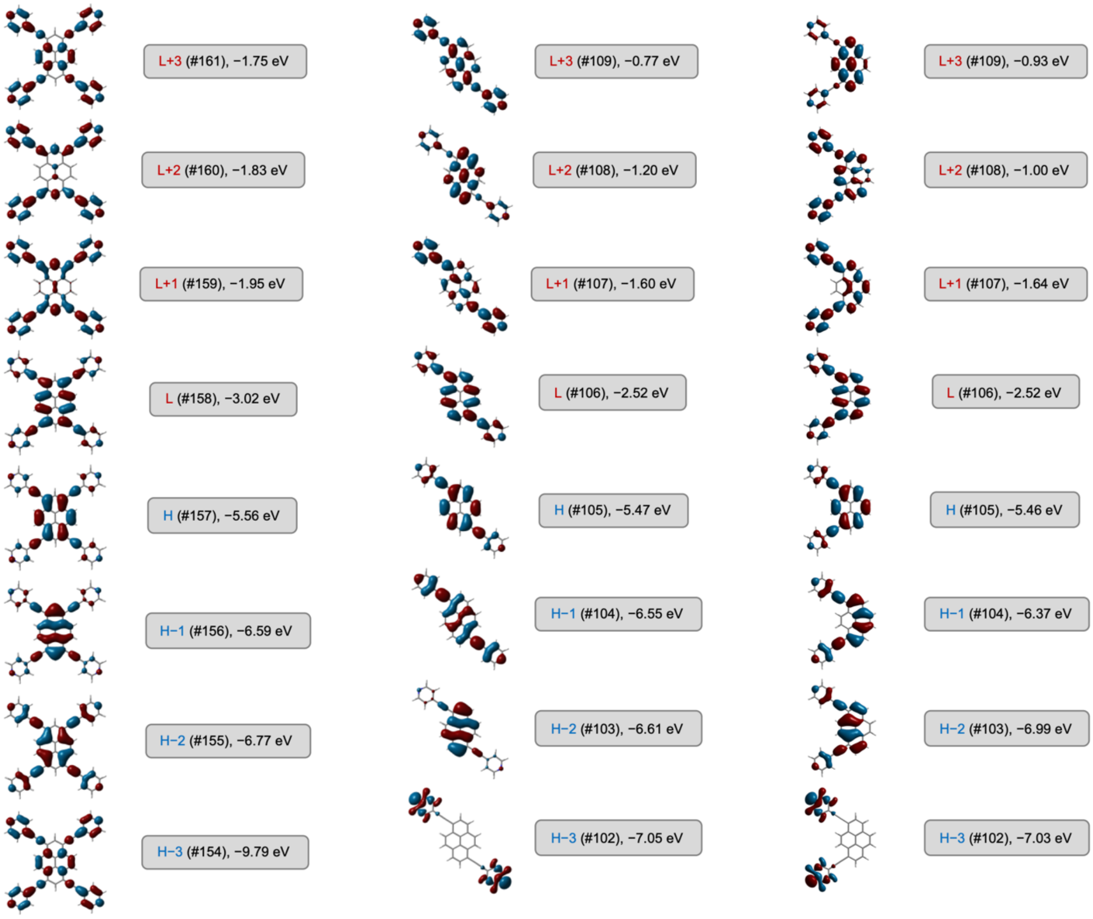

Figure S2. Frontier molecular orbitals (isovalue contours ± 0.02 a.u.) for **1** (left), **2** (center) and **3** (right) from B3LYP/6-31G(d,p) calculations.

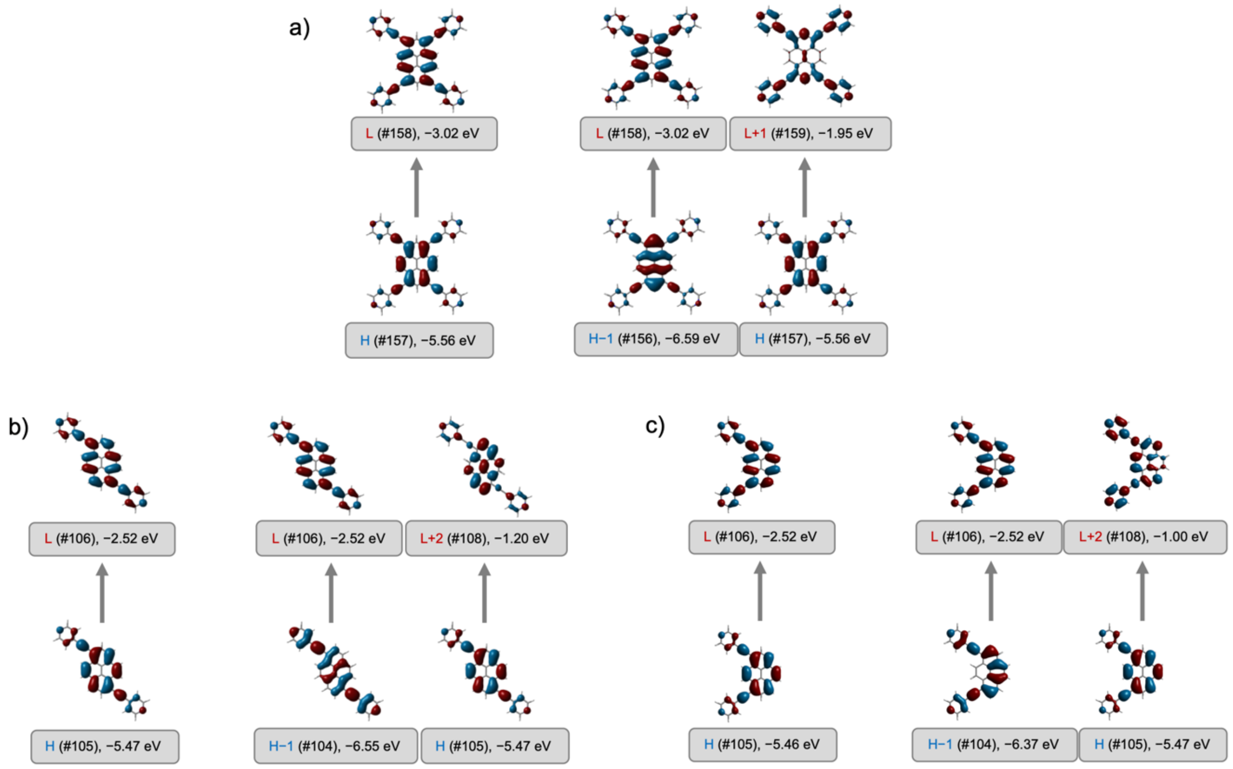

Figure S3. Dominant orbital contributions to the first and second excitations in pyrenes (a) **1**, (b) **2**, and (c) **3**, obtained from DFT calculations at the B3LYP/6-31G(d,p) level of theory.

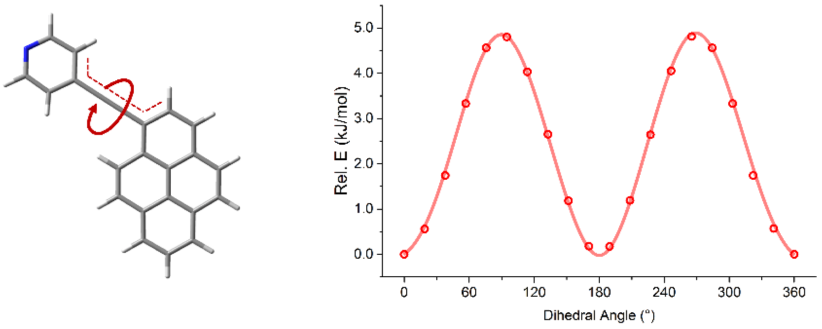

Figure S4. Model pyridylethynyl pyrene compound used for calculating the energy profile for the rotation around the triple bond, at the B3LYP/6-31G(d,p) level of theory.

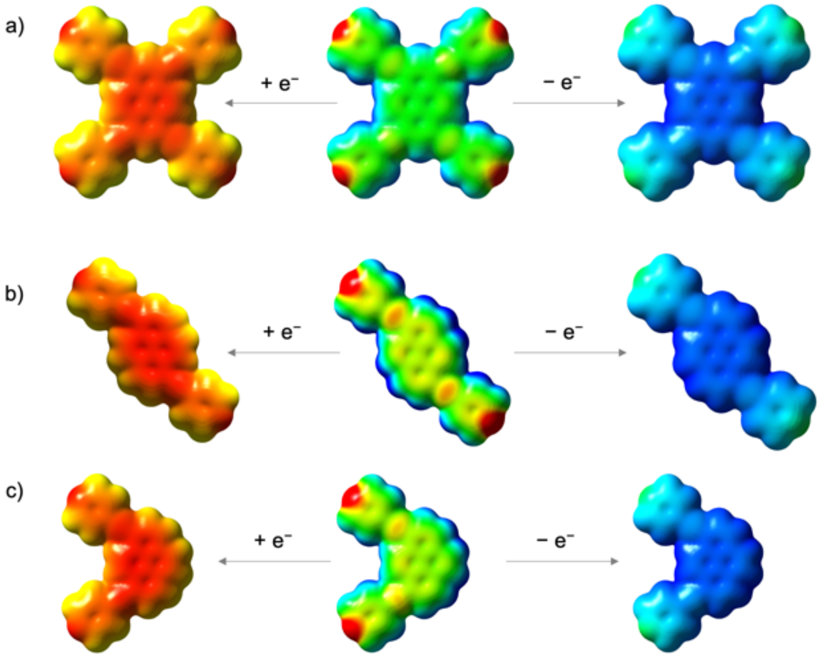

Figure S5. Mapped electrostatic potential of (a) tetrasubstituted pyrene **1**, (b) *trans*-like pyrene **2** and (c) *cis*-like pyrene **3**.

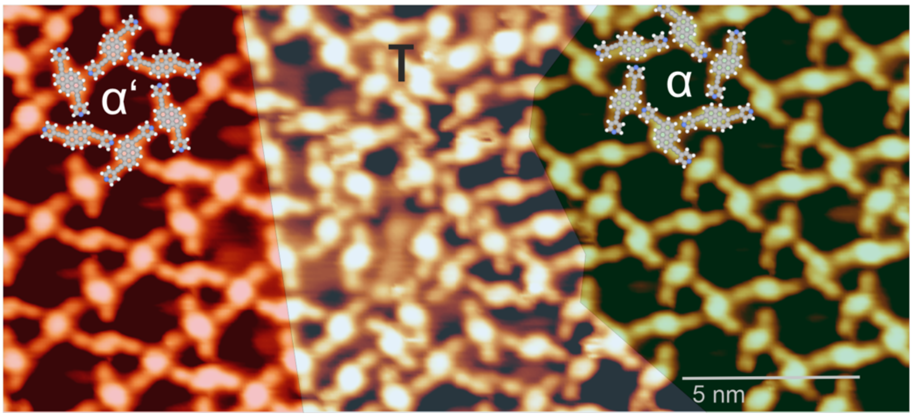

Figure S6. STM image of homo-chiral mirror domains coexisting in a sub-monolayer of *trans*-like pyrene **2** on *h*BN/Cu(111). The two domains are labeled *α'* and *α*, respectively, with a transition region *τ*. Overlaid structural models sketch the molecular assembly. Imaging parameters: 14 x 25 nm, 1.04 V, 0.1 nA.

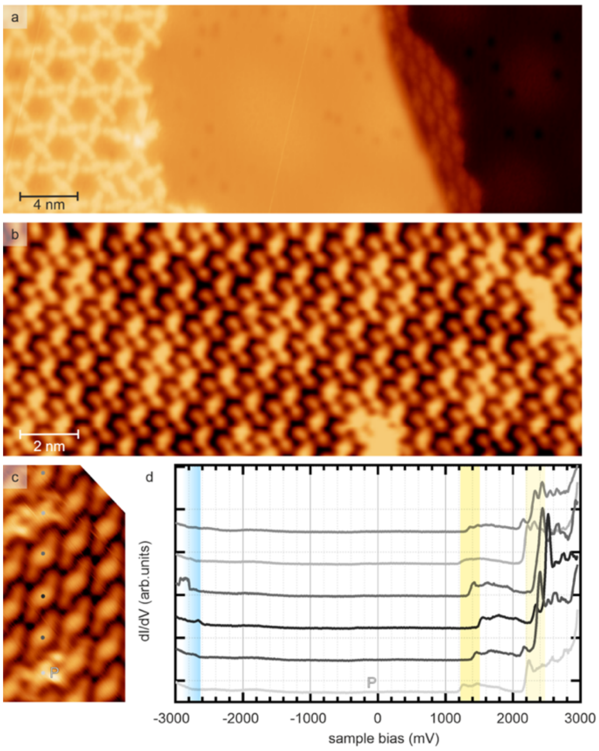

Figure S7. STM and STS data of dense-packed islands of *trans*-like derivative **2**. (a) Dense-packed array at a step edge, coexisting with the kagomé-like network (1.04 V, 0.05 nA). (b) Extended, homo-chiral island with modulated apparent height induced by the moiré pattern of the underlying hBN/Cu(111) (1.21 V, 0.035 nA). (c) STM image with grey markers indicating the lateral positions of the dI/dV spectra shown in (d) (1.296 V, 0.08 nA). (d) Vertically offset dI/dV spectra near pore (P) and wire positions, with the energy range of HOMO, LUMO, and LUMO+1 onsets highlighted by blue, yellow, and light yellow bars, respectively (tip stabilization: -2.62 V, 0.08 nA) On the wire area, the HOMO is observed at -2.69 V and the LUMO occurs at 1.37 V, yielding an STM-derived HOMO-LUMO gap of 4.06 eV. Compare Figures 5 and 6 in the main text.

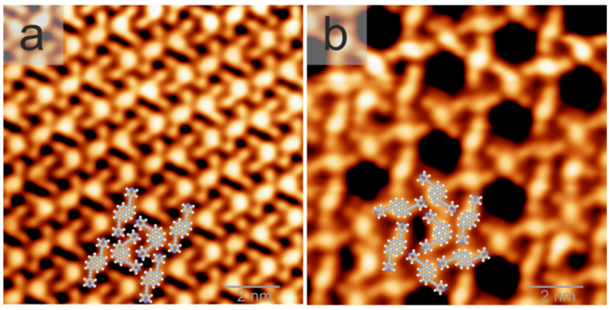

Figure S8. Binary assemblies from *cis-* and *trans*-like pyrene derivatives **2** and **3** on hBN/Cu(111). Sequential dosing of species **3** and **2** at RT yields two coexisting assemblies, namely (a) dense-packed binary islands (where dimer rows of derivative **3** are separated by rows of derivative **2**) and (b) a kagomé lattice derived from derivative **2** hosting species **3** in the large pores. The *cis*-like guests occur in six distinct rotational alignments, three of which are discernible in (b). (1 V, 0.06 nA).

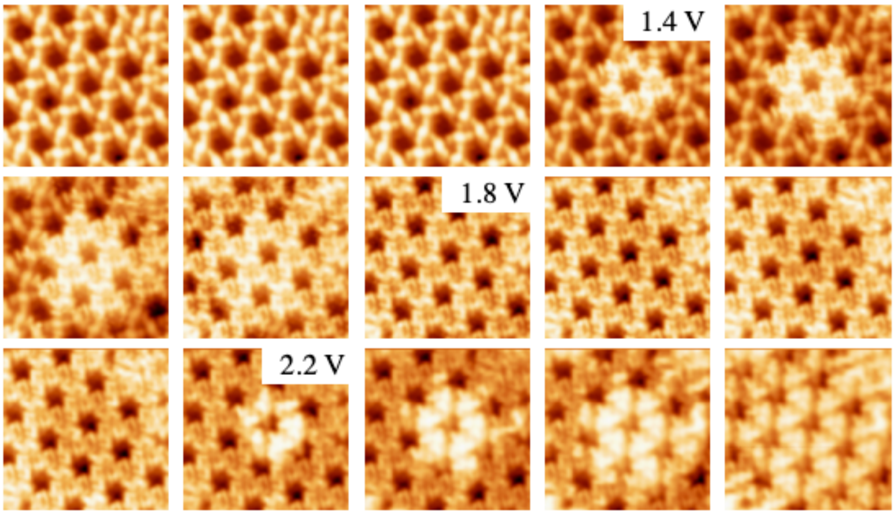

Figure S9. STM image series of the kagomé-like network of *trans*-like pyrene **2** recorded increasing the bias voltage from 1.1 V to 2.5 V in steps of 0.1 V. Crossing the energies of the LUMO and LUMO+1 states yields distinct changes in the apparent height and in the sub-molecular contrast. These changes first appear on the pore regions (see images at 1.4 and 2.2 V). At intermediate bias voltages (e.g., image at 1.8 V) no pronounced difference between pore and wire areas is discernible. See also movie provided as additional file in the Supporting Information. (Image size: 11 nm x 11 nm).

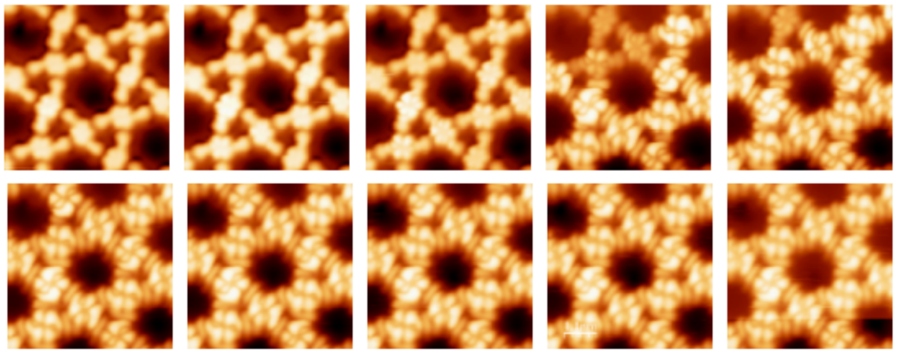

Figure S10. STM image series network of *trans*-like pyrene **2** recorded increasing the bias voltage from -1.8 V to -2.7 V in steps of 0.1 V. As for the unoccupied states (see Figure S9), the apparent height and the sub-molecular contrast changes considerably when the HOMO resonance is accessed. (Image size: 11 nm x 11 nm).

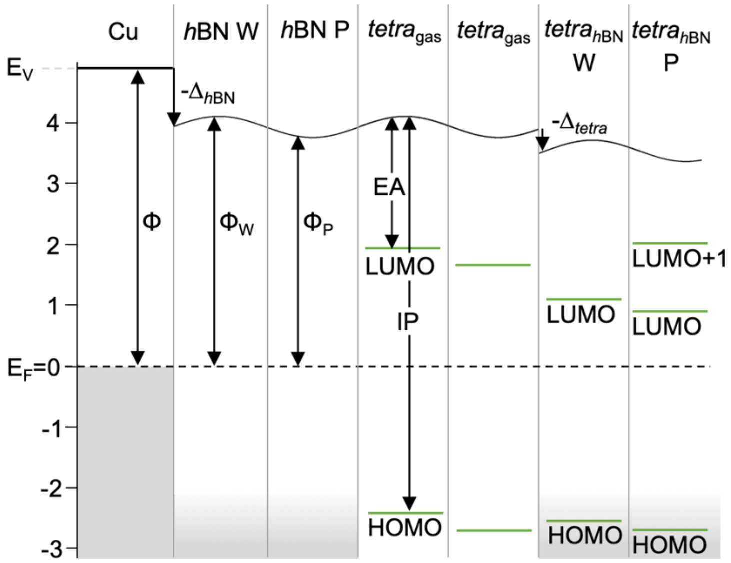

Figure S11. Sketch of the energy level alignment in the tetrasubstituted pyrene/hBN/Cu(111) system, separated for Cu, bare *h*BN/Cu, tetrasubstituted pyrene **1** in gas phase, and adsorbed on hBN/Cu. Relevant parameters are included (ϕ: work function Cu(111), ϕ_W: local work function of wire area (W) of the *h*BN moiré, ϕ_P: local work function of pore area (P) of the *h*BN moiré, EA / IP: calculated electron affinity and ionization potential of pyrene **1** in gas phase). The MOs are aligned to the local vacuum level (E_V) and thus shift relative to the Fermi level (E_F). Note that the indicated change in local vacuum level upon molecular adsorption ($-\Delta_{tetra}$) reflects a tentative assignment.

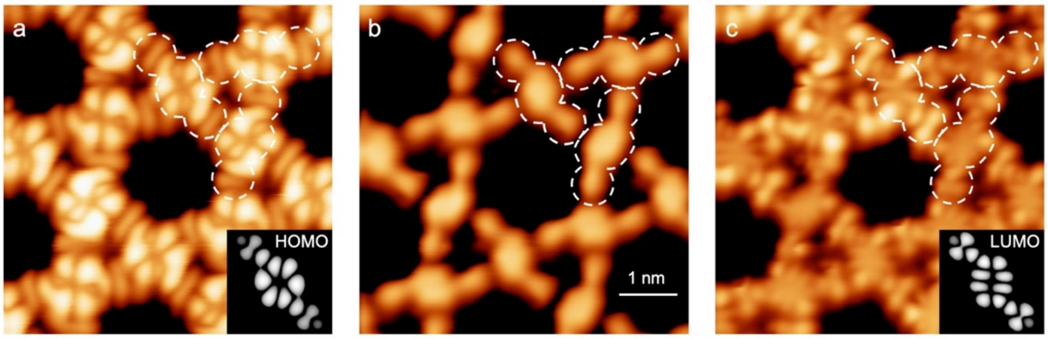

Figure S12. Bias-dependent intramolecular contrast in the kagomé-like network of pyrene derivative **2** reflecting (a) HOMO and (c) LUMO-like structures at -2.4 and 2.1 V, respectively. (b) In-gap image recorded at 1.2 V. The dashed outlines highlight molecules in the three different orientations. The insets show EHT-calculated HOMO (in (a)) and LUMO (in (c)) of the free molecule. Compare Figure 7 in the main text. (Image size: 5.54 nm x 5.54 nm, 0.2 nA).

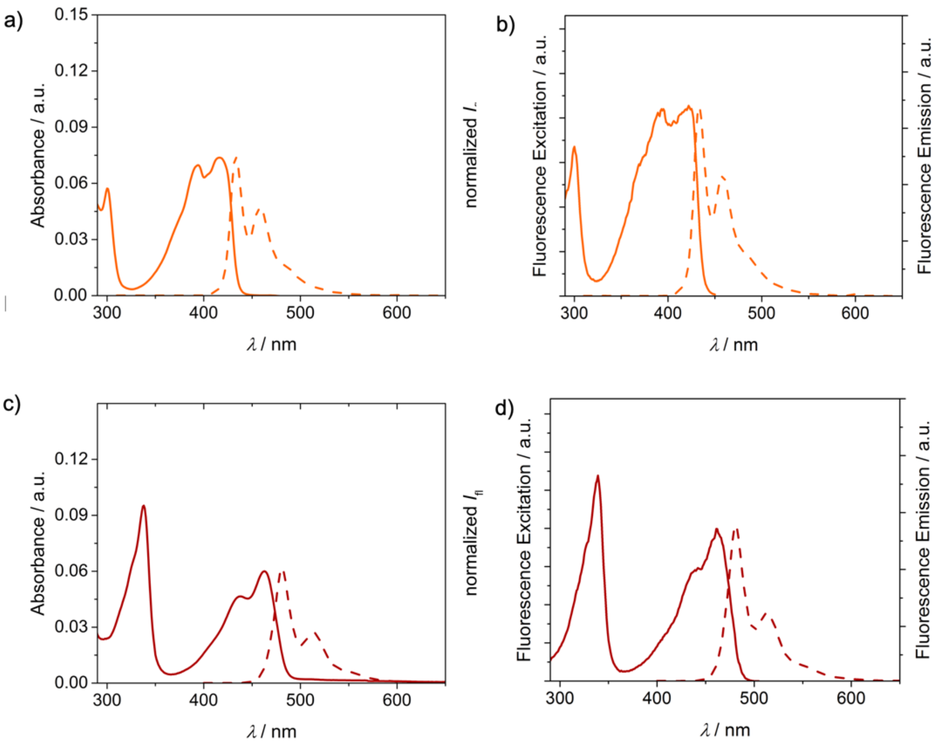

Figure S13. Absorption and emission spectra for pyrenes **1** (top) and **2** (bottom). Panels (a) and (c) are absorbance and emission spectra, while panels (b) and (d) were obtained by recording the fluorescence excitation.

Table S1. First 10 electronic transition calculated for tetrasubstituted pyrene **1** using TD-CAM-B3LYP/6-31G** level of theory.

No.	Energy (cm^{-1})	λ (nm)	f[a]	Major excitations[b]
**1**	21197.9	471.7	1.60	H → L (96%)
**2**	27997.9	357.2	0.04	H-1 → L (53%), H → L+1 (26%), H → L+4 (10%)
**3**	30944.3	323.2	2.86	H-1 → L (32%), H → L+1 (54%)
**4**	31561.3	316.8	0	H-3 → L (42%), H → L+2 (39%)
**5**	32826.0	304.6	0	H-2 → L (71%), H → L+3 (17%)
**6**	33100.2	302.1	0	H-13 → L (15%), H-3 → L (33%), H → L+2 (38%)
**7**	33984.2	294.3	0	H-2 → L (20%), H → L+3 (71%)
**8**	35947.3	278.2	0	H-13 → L (71%), H → L+6 (14%)
**9**	37785.5	264.7	0	H-16 → L+3 (11%), H-15 → L (67%)
**10**	38258.9	261.4	0	H-17 → L+2 (10%), H-16 → L (62%), H-15 → L+3 (12%)

[a] Oscillator strength; [b] H = HOMO, L = LUMO, contributions smaller than 10% are not reported.

Table S2. First 10 electronic transition calculated for *trans*-like pyrene **2** using TD-CAM-B3LYP/6-31G** level of theory.

No.	Energy (cm^{-1})	λ (nm)	f[a]	Major excitations[b]
1	24335.4	410.9	1.82	H → L (95%)
2	30181.3	331.3	0.02	H-1 → L (55%)<br>H → L+2 (31%)
3	32617.1	306.6	0	H-2 → L (31%)<br>H → L+1 (58%)
4	35243.2	283.7	0	H-3 → L (10%)<br>H-2 → L (50%),<br>H → L+1 (26%)
5	36703.1	272.5	1.10	H-1 → L (32%),<br>H → L+2 (53%)
6	37558.0	266.3	0	H-3 → L (69%),<br>H → L+4 (19%)
7	38704.1	258.4	0	H-10 → L+1 (26%),<br>H-9 → L (66%)
8	38913.8	257.0	0	H-10 → L (56%),<br>H-9 → L+1 (25%)
9	38955.8	256.7	0.01	H-7 → L+1 (37%),<br>H-6 → L (32%),<br>H-6 → L+3 (17%)
10	38960.6	256.7	0	H-7 → L (28%),<br>H-7 → L+3 (15%),<br>H-6 → L+1 (33%)

[a] Oscillator strength; [b] H = HOMO, L = LUMO, contributions smaller than 10% are not reported.

Table S3. First 10 electronic transition calculated for *cis*-like pyrene **3** using TD-CAM-B3LYP/6-31G** level of theory.

No.	Energy (cm^{-1})	λ (nm)	f[a]	Major excitations[b]
1	24328.9	411.0	1.25	H → L (95%)
2	29894.1	334.5	0.01	H-2 → L (11%)<br>H-1 → L (43%)<br>H → L+1 (18%)<br>H → L+3 (21%)
3	32296.1	309.6	1.49	H-1 → L (34%)<br>H → L+1 (55%)
4	36549.8	273.6	0.01	H-6 → L (23%)<br>H-3 → L (62%)
5	37395.1	267.4	0.01	H-2 → L (36%)<br>H-1 → L (13%)<br>H → L+1 (18%)<br>H → L+3 (24%)
6	37412.0	267.3	0.01	H → L+2 (51%)<br>H → L+4 (30%)
7	38691.2	258.5	0	H-10 → L+1 (25%)<br>H-9 → L (65%)
8	38835.6	257.5	0	H-10 → L (63%)<br>H-9 → L+1 (26%)
9	38938.0	256.8	0	H-8 → L (32%)<br>H-8 → L+2 (20%)<br>H-7 → L+1 (31%)
10	38938.8	256.8	0.01	H-8 → L+1 (32%)<br>H-7 → L (32%)<br>H-7 → L+2 (20%)

[a] Oscillator strength; [b] H = HOMO, L = LUMO, contributions smaller than 10% are not reported.

Table S4. Cartesian coordinates of optimized tetrasubstituted pyrene **1** at the B3LYP/6-31G** level of theory.

Atom	x	y	z
C	5.9980	-3.0430	5.3560
C	7.3770	-3.0040	5.1510
C	7.8970	-2.5850	3.9280
C	7.0350	-2.1910	2.8770
C	7.5190	-1.7590	1.6300
C	6.6560	-1.3750	0.6060
C	5.2610	-1.4020	0.7710
C	4.3700	-1.0170	-0.2590
C	2.9920	-1.0610	-0.0560
C	2.4710	-1.4840	1.1660
C	3.3330	-1.8740	2.2190
C	2.8480	-2.3050	3.4660
C	3.7120	-2.6860	4.4910
C	5.1070	-2.6580	4.3260
C	5.6280	-2.2280	3.0760
C	4.7400	-1.8340	2.0210
C	5.5410	-3.4880	6.6430
C	4.8350	-0.5710	-1.5430
C	5.2230	-0.1960	-2.6160
C	5.1580	-3.8540	7.7220
C	4.7020	-4.2930	9.0030
C	5.6830	0.2450	-3.8960
C	7.0470	0.3060	-4.1660
C	7.4410	0.7360	-5.4210
N	6.5790	1.0980	-6.3960
C	5.2630	1.0280	-6.1030
C	4.7680	0.6110	-4.8790
C	5.6180	-4.6600	9.9840
C	5.1260	-5.0790	11.2080
N	3.8100	-5.1510	11.5030
C	2.9460	-4.7890	10.5300
C	3.3380	-4.3560	9.2750
C	9.3270	-2.5840	3.7900
C	10.5220	-2.5830	3.6700
C	11.9450	-2.5730	3.5280
C	1.0410	-1.5130	1.3000
C	-0.1550	-1.5320	1.4090
C	-1.5790	-1.5580	1.5340
C	12.7550	-2.9760	4.5860
C	14.1260	-2.9450	4.4000
N	14.7200	-2.5470	3.2550
C	13.9070	-2.1630	2.2470
C	12.5250	-2.1580	2.3340
C	-2.3810	-1.1700	0.4650
C	-3.7540	-1.2150	0.6350
N	-4.3570	-1.6130	1.7760
C	-3.5510	-1.9820	2.7950
C	-2.1690	-1.9730	2.7240
H	8.0510	-3.3030	5.9520
H	8.5890	-1.7140	1.4310
H	7.1020	-1.0510	-0.3330
H	2.3170	-0.7640	-0.8580
H	1.7790	-2.3520	3.6640
H	3.2660	-3.0090	5.4300
H	7.7870	0.0270	-3.4240
H	8.4930	0.8020	-5.6850
H	4.5930	1.3250	-6.9050
H	3.6990	0.5760	-4.7020
H	6.6870	-4.6230	9.8060
H	5.7970	-5.3760	12.0090
H	1.8950	-4.8570	10.7960
H	2.5970	-4.0780	8.5350
H	12.3360	-3.3050	5.5300
H	14.8050	-3.2480	5.1920
H	14.4120	-1.8490	1.3390
H	11.9240	-1.8370	1.4900
H	-1.9550	-0.8420	-0.4760
H	-4.4270	-0.9240	-0.1670
H	-4.0640	-2.2970	3.6990
H	-1.5740	-2.2810	3.5770

Table S5. Cartesian coordinates of optimized *trans*-like pyrene **2** at the B3LYP/6-31G** level of theory.

Atom	x	y	z
C	2.9100	1.0680	0.0010
C	2.5550	2.4330	0.0010
C	1.2280	2.8270	0.0020
C	0.1900	1.8780	0.0010
C	-1.1900	2.2580	0.0010
C	-2.1840	1.3250	0.0010
C	-1.8880	-0.0770	0.0010
C	-2.9100	-1.0680	0.0010
C	-2.5550	-2.4330	0.0010
C	-1.2280	-2.8270	0.0020
C	-0.1900	-1.8780	0.0010
C	1.1900	-2.2580	0.0010
C	2.1840	-1.3250	0.0010
C	1.8880	0.0770	0.0010
C	0.5210	0.4890	0.0010
C	-0.5210	-0.4890	0.0010
C	4.2830	0.7070	0.0000
C	-4.2830	-0.7070	0.0000
C	-5.4660	-0.4170	-0.0010
C	5.4660	0.4170	-0.0010
C	6.8480	0.0840	-0.0010
C	-6.8480	-0.0840	-0.0010
C	-7.8400	-1.0820	-0.0020
C	-9.1780	-0.7000	-0.0020
N	-9.5960	0.5730	-0.0020
C	-8.6470	1.5200	-0.0020
C	-7.2810	1.2550	-0.0020
C	7.8400	1.0820	-0.0020
C	9.1780	0.7000	-0.0020
N	9.5960	-0.5730	-0.0020
C	8.6470	-1.5200	-0.0020
C	7.2810	-1.2550	-0.0020
H	3.3460	3.1760	0.0010
H	0.9770	3.8840	0.0020
H	-1.4350	3.3160	0.0020
H	-3.2250	1.6300	0.0010
H	-3.3460	-3.1760	0.0010
H	-0.9770	-3.8840	0.0020
H	1.4350	-3.3160	0.0020
H	3.2250	-1.6300	0.0010
H	-7.5620	-2.1310	-0.0010
H	-9.9580	-1.4590	-0.0030
H	-8.9990	2.5500	-0.0030
H	-6.5600	2.0660	-0.0020
H	7.5620	2.1310	-0.0010
H	9.9580	1.4590	-0.0030
H	8.9990	-2.5500	-0.0030
H	6.5600	-2.0660	-0.0020

Table S6. Cartesian coordinates of optimized *cis*-like pyrene **3** at the B3LYP/6-31G** level of theory.

Atom	x	y	z
C	-2.8300	3.8070	0.0000
C	-3.5280	2.6100	0.0000
C	-2.8530	1.3730	0.0000
C	-1.4290	1.3510	0.0000
C	-0.6820	0.1300	0.0000
C	0.6820	0.1300	0.0000
C	1.4290	1.3510	0.0000
C	2.8530	1.3730	0.0000
C	3.5270	2.6100	0.0000
C	2.8300	3.8070	0.0000
C	1.4240	3.8260	0.0000
C	0.6810	5.0520	0.0000
C	-0.6810	5.0520	0.0000
C	-1.4240	3.8260	0.0000
C	-0.7150	2.5860	0.0000
C	0.7150	2.5860	0.0000
C	3.6090	0.1700	0.0000
C	4.2790	-0.8460	0.0000
C	5.0700	-2.0280	0.0000
C	4.4820	-3.3070	0.0000
C	5.3070	-4.4270	-0.0010
N	6.6470	-4.3740	-0.0010
C	7.2020	-3.1540	-0.0010
C	6.4770	-1.9660	0.0000
C	-3.6090	0.1700	0.0000
C	-4.2790	-0.8460	0.0000
C	-5.0700	-2.0280	0.0000
C	-6.4770	-1.9660	-0.0040
C	-7.2010	-3.1540	-0.0040
N	-6.6460	-4.3740	0.0000
C	-5.3070	-4.4270	0.0040
C	-4.4820	-3.3070	0.0040
H	-4.6120	2.6110	0.0000
H	-1.2290	-0.8070	0.0000
H	1.2290	-0.8070	0.0000
H	4.6120	2.6110	0.0000
H	1.2320	5.9890	0.0000
H	-1.2320	5.9890	0.0000
H	3.4030	-3.4150	0.0000
H	4.8680	-5.4230	-0.0010
H	8.2900	-3.1240	-0.0010
H	6.9830	-1.0080	0.0000
H	-6.9830	-1.0080	-0.0080
H	-8.2890	-3.1240	-0.0070
H	-4.8680	-5.4230	0.0070
H	-3.4020	-3.4150	0.0080
H	3.3720	4.7480	0.0000
H	-3.3720	4.7480	0.0000
